# CKB Promotes Mitochondrial ATP Production by Suppressing Permeability Transition Pore

**DOI:** 10.1002/advs.202403093

**Published:** 2024-06-19

**Authors:** Le He, Jianghua Lin, Shaojuan Lu, Hao Li, Jie Chen, Xinyi Wu, Qixin Yan, Hailiang Liu, Hui Li, Yufeng Shi

**Affiliations:** ^1^ Tongji University Cancer Center Shanghai Tenth People's Hospital of Tongji University School of Medicine Tongji University Shanghai 200092 China; ^2^ State Key Laboratory of Cardiology and Medical Innovation Center Shanghai East Hospital School of Medicine Tongji University Shanghai 200123 China; ^3^ Key Laboratory of Spine and Spinal Cord Injury Repair and Regeneration of Ministry of Education Tongji University Cancer Center Shanghai Tenth People's Hospital of Tongji University School of Medicine Tongji University Shanghai 200092 China

**Keywords:** aging, AKT, cancer, creatine kinase brain‐type (CKB), F1F0 ATP synthase, mitochondrial permeability transition pore (mPTP)

## Abstract

Creatine kinases are essential for maintaining cellular energy balance by facilitating the reversible transfer of a phosphoryl group from ATP to creatine, however, their role in mitochondrial ATP production remains unknown. This study shows creatine kinases, including CKMT1A, CKMT1B, and CKB, are highly expressed in cells relying on the mitochondrial F1F0 ATP synthase for survival. Interestingly, silencing CKB, but not CKMT1A or CKMT1B, leads to a loss of sensitivity to the inhibition of F1F0 ATP synthase in these cells. Mechanistically, CKB promotes mitochondrial ATP but reduces glycolytic ATP production by suppressing mitochondrial calcium (mCa^2+^) levels, thereby preventing the activation of mitochondrial permeability transition pore (mPTP) and ensuring efficient mitochondrial ATP generation. Further, CKB achieves this regulation by suppressing mCa^2+^ levels through the inhibition of AKT activity. Notably, the CKB‐AKT signaling axis boosts mitochondrial ATP production in cancer cells growing in a mouse tumor model. Moreover, this study also uncovers a decline in CKB expression in peripheral blood mononuclear cells with aging, accompanied by an increase in AKT signaling in these cells. These findings thus shed light on a novel signaling pathway involving CKB that directly regulates mitochondrial ATP production, potentially playing a role in both pathological and physiological conditions.

## Graphical abstract

CKB is highly expressed and necessary for maintaining cell sensitivity to the blockage of F1F0 ATP synthase. CKB and phosphocreatine, suppress AKT activation. CKB‐AKT signaling inhibits mitochondrial calcium levels, which in turn inactivates the mPTP. CKB‐AKT signaling is involved in the mitochondrial energy metabolism of tumor progression. AKT activation resulting from declined CKB likely leads to dysregulated mitochondrial energy production in Aging.

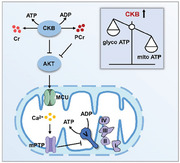

## 1 Introduction

Creatine kinases (CKs), also known as creatine phosphokinases,^[^ ^1^ ^]^ are a family of enzymes comprising multiple different isoforms, including creatine kinase ubiquitous‐type CKMT1 (mitochondria), creatine kinase sarcomeric‐type CKMT2 (mitochondria), creatine kinase brain‐type CKB (mainly cytoplasm), and creatine kinase muscle‐type CKM (cytoplasm).^[^ ^1^, ^2^ ^]^ These CK isoforms are distributed across various tissues in the body, and play vital roles in cellular energy metabolism, particularly in tissues with high and fluctuating energy demands, such as skeletal muscles, the heart, the brain, the immune system, and cancers.^[^ ^1^, ^2^, ^3^ ^]^ Studies have highlighted the significance of altered expression of these CK isoforms in various tissues and cancers, with changes in CK levels often linked to tumor progression.^[^ ^4^ ^]^

The primary function of CKs is to facilitate the reversible transfer of phosphate groups between ATP and creatine, leading to the production of phosphocreatine and adenosine diphosphate (ADP). This enzymatic reaction is crucial for preserving cellular energy balance, enabling the rapid generation of ATP during times of increased energy requirements.^[^ ^1a^ ^]^ Apart from their well‐established function in energy homeostasis, CKs have also been implicated in noncanonical roles in various cellular processes, such as ferroptosis.^[^ ^5^ ^]^ Despite these findings, the specific involvement of CKs in mitochondrial energy production remains unclear and requires further investigation.

Mitochondrial oxidative phosphorylation (OXPHOS) is a critical process in cellular energy metabolism. The OXPHOS electron transport chain (ETC) consists of four complexes (I to IV) that facilitate the transfer of electrons from NADH or FADH_2_ produced in the TCA cycle and fatty acid oxidation to oxygen.^[^ ^6^ ^]^ The energy generated by the ETC is stored in the mitochondrial membrane potential (MMP), which is then harnessed by the F1F0 ATP synthase within OXPHOS to synthesize ATP.^[^ ^6b^ ^,7]^ The activity of mitochondrial OXPHOS is tightly regulated by various upstream signaling pathways, including AKT signaling, AMPK signaling, hypoxia, and others.^[^ ^8^ ^]^ AKT signaling, in particular, has been shown to influence mitochondrial function through diverse mechanisms such as moderation of mitochondrial metabolism, regulation of mitochondrial calcium (mCa^2+^) homeostasis, and promotion of mitogenesis.^[^ ^9^ ^]^ Notably, AKT activation has been observed to be upregulated in aging tissues,^[^ ^10^ ^]^ suggesting a potential link between AKT signaling and age‐related changes in mitochondrial function.

The mitochondrial permeability transition pore (mPTP) is a nonselective mega channel located in the inner mitochondrial membrane, and its exact protein composition remains unknown.^[^ ^11^ ^]^ The mPTP plays a critical role in regulating mitochondrial membrane permeability, allowing the passage of small molecules with a molecular weight below ≈1.5 kDa in and out of the mitochondria, leading to disrupted MMP, reduced efficiency of mitochondrial ATP production, and other mitochondrial dysfunctions.^[^ ^12^ ^]^ The activity of mPTP is often upregulated in aged tissues and cells, and multiple factors are involved in the regulation of mPTP activity, including mCa^2+^, reactive oxidative species (ROS), and others.^[^ ^12^, ^13^ ^]^ The complex regulation of mPTP also involves specific proteins such as the adenine nucleotide translocase (ANT) and cyclophilin D (CypD).^[^ ^14^ ^]^ Notably, cyclosporine A (CsA), a well‐known inhibitor of mPTP, targets CypD and thus blocks mPTP activity.^[^ ^14b,c^ ^]^

In this study, we discovered high expression of CKs in cells, which rely on mitochondrial ATP for survival. CKB suppresses AKT activation, leading to decreased mCa^2+^ levels and mPTP activity, ultimately resulting in efficient mitochondrial ATP production. Our data also implies that CKB's regulation of mitochondrial ATP generation is involved in cancer progression and aging.

## 2 Result

### 2.1 High Expression of Creatine Kinases in Cells Susceptible to F1F0 ATP Synthase Inhibition

**Figure 1:**
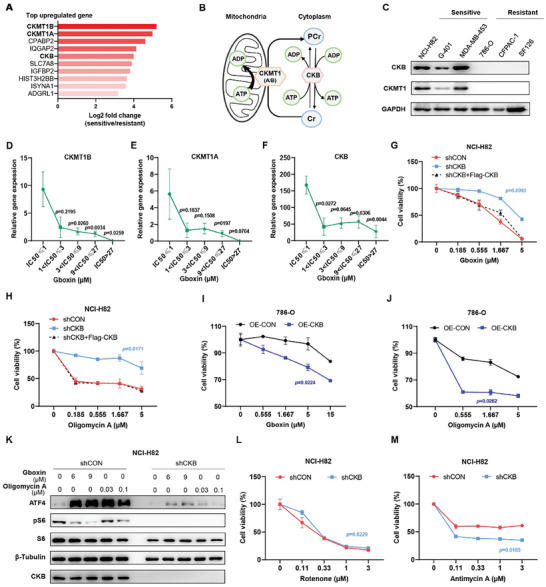
CKB plays a pivotal role in determining cellular susceptibility to F1F0 ATP synthase inhibition. A) Examination of differentially expressed genes (DEGs) reveals heightened expression of creatine metabolism‐related genes in Gboxin‐sensitive cell lines (NCI‐H82, G‐401, and MDA‐MB‐453) compared to Gboxin‐resistant cell lines (786‐O, CFPAC‐1, and SF126). B) Illustration of the CK/phosphocreatine system's role in cellular energy shuttling. PCr: phosphocreatine; Cr: creatine. C) Immunoblot analysis of CKMT1 and CKB in Gboxin‐sensitive and Gboxin‐resistant cell lines, with GAPDH serving as a loading control. *n* = 3. D–F) Transcription of CKMT1B (D), CKMT1A (E), and CKB (F) positively correlates with cell sensitivity to Gboxin treatment. The 57 cancer cell lines were categorized into five groups based on their IC_50_ in Gboxin sensitivity screening. Gene transcription data was sourced from the Cancer Cell Line Encyclopedia (CCLE). Mean ± SEM. *p* values determined by one‐way ANOVA. G–J) Cell viability analyses demonstrate the critical role of CKB in maintaining cell sensitivity to the inhibition of F1F0 ATP synthase. The Cell Titer‐Glo reagent was used to detect the cell viability of NCI‐H82‐shCON (G,H), NCI‐H82‐shCKB (G,H), NCI‐H82‐shCKB+Flag‐CKB (G,H), 786O‐OE‐CON (I,J), and 786O‐OE‐CKB (I,J) incubated with Gboxin (G,I) or Oligomycin A (H,J) at various concentrations for 3 days. Mean ± SD. *n* = 3. Paired *t*‐test reveals the lowest *p* value compared to the vector. K) Western blot analysis demonstrates a reduced upregulation of the mitochondrial stress‐responding protein ATF4 in NCI‐H82‐shCKB cells compared to NCI‐H82‐shCON cells. Cells were treated with DMSO, Gboxin, or Oligomycin A at the indicated concentrations for 3 h. *n* = 3. L,M) Cell viability analyses reveal no difference in Rotenone sensitivity between NCI‐H82‐shCON and NCI‐H82‐shCKB cells (L). In contrast, cell viability analyses show enhanced sensitivity of NCI‐H82‐shCKB cells to Antimycin A treatment compared to NCI‐H82‐shCON cells (M). Cells were incubated with Rotenone (L) and Antimycin A (M) at various concentrations indicated for 3 days. Mean ± SD. *n* = 3. Paired *t*‐test indicates the lowest *p*‐value compared to the vector.

In a recent study, we performed a sensitivity screen using Gboxin,^[^ ^15^ ^]^ a newly identified inhibitor of F1F0 ATP synthase, on 57 cancer cell lines representing various tumor types. Our results showed varying sensitivities to Gboxin treatment among the tested cancer cell lines.^[^ ^15^ ^]^ We further validated these screening outcomes and selected Gboxin‐sensitive cell lines (NCI‐H82, G‐401, and MDA‐MB‐453) as well as Gboxin‐resistant cell lines (786‐O, CFPAC‐1, and SF126) for differential pathway and gene expression analyses. Our data revealed that genes involved in creatine metabolism, such as CKMT1A, CKMT1B, and CKB, were significantly upregulated in Gboxin‐sensitive cell lines compared to their resistant counterparts (**Figure** **1**A; Figure S1A–C, Supporting Information). Notably, CKMT1A and CKMT1B encode the same mitochondrial enzyme CKMT1, which facilitates the transfer of a phosphate group from mitochondrial ATP to creatine, generating phosphocreatine released into the cytoplasm. On the other hand, CKB primarily catalyzes the interconversion between phosphocreatine and creatine in the cytoplasm (Figure 1B).^[^ ^1^, ^16^ ^]^

The expression of CKMT1 (encoded by CKMT1A and CKMT1B) and CKB in Gboxin‐sensitive (NCI‐H82, G‐401 and MDA‐MB‐453) and Gboxin‐resistant (786‐O, CFPAC‐1 and SF126) cell lines was confirmed by immunoblot analysis, revealing elevated levels of CKMT1 and CKB proteins in Gboxin‐sensitive cells and undetectable levels in Gboxin‐resistant cells (Figure 1C). Additionally, we categorized the 57 cancer cell lines from the Gboxin sensitivity screen into five groups based on their sensitivities, illustrating a positive correlation between the expression of CKMT1A, CKMT1B, and CKB and the cell's sensitivity to Gboxin treatment (Figure 1C–F). Moreover, leveraging gene expression and drug sensitivity data from the Cancer Dependency Map Project (Depmap) at the Broad Institute, we identified strong positive associations between the expression of CKMT1A, CKMT1B, and CKB, and the cell's sensitivity to Oligomycin A, another inhibitor of F1F0 ATP synthase (Figure S1D–F, Supporting Information). These findings suggest a significant link between the expression of key enzymes in creatine metabolism and the cell's sensitivity to F1F0 ATP synthase inhibition, indicating a potential interplay between creatine kinases and mitochondrial ATP production.

### 2.2 CKB is Essential for Maintaining Cellular Sensitivity to F1F0 ATP Synthase Inhibition

To investigate the impact of creatine kinases on cellular sensitivity to F1F0 ATP synthase inhibition, we manipulated the expression of creatine kinase genes in both Gboxin‐sensitive and ‐resistant cell lines. Surprisingly, targeted deletion of mitochondrial CKMT1 by CRISPR/Cas9‐mediated knocking out of both CKMT1A and CKMT1B genes did not lead to discernible changes in the cells’ responsiveness to F1F0 ATP synthase inhibition by Gboxin or Oligomycin A (Figure S2A–D, Supporting Information). Furthermore, the overexpression of CKMT1 in Gboxin‐resistant cells did not alter the cell's sensitivity to these inhibitors (Figure S2E–G, Supporting Information).

We then explored the potential role of CKB in modulating cell sensitivity to F1F0 ATP synthase inhibition. As shown in Figure 1G,H; Figure S2H–J (Supporting Information), knocking down CKB in NCI‐H82 cells (NCI‐H82‐shCKB cells and NCI‐H82‐shCKB‐2 cells) resulted in resistance to F1F0 ATP synthase inhibitors, Gboxin and Oligomycin A. To rule out potential off‐target effects from CKB shRNAs, we reintroduced CKB in NCI‐H82‐shCKB cells (Figure S2K, Supporting Information), which restored their sensitivity to Gboxin or Oligomycin A treatment (Figure 1G,H). Additionally, as depicted in Figure 1I,J; Figure S2L (Supporting Information), overexpressing of CKB in Gboxin‐resistant 786‐O cells increases their sensitivity to F1F0 ATP synthase inhibition compared to control cells. These results suggest that CKB plays a crucial role in maintaining cell sensitivity to F1F0 ATP synthase inhibition within the OXPHOS pathway. Inhibition of OXPHOS complexes often triggers the activation of the ATF4 stress response pathway.^[^ ^17^ ^]^ Consistent with the decreased sensitivity of NCI‐H82 cells to F1F0 ATP synthase inhibition following CKB deletion, inhibiting CKB significantly attenuated ATF4 upregulation upon Gboxin or Oligomycin A treatment (Figure 1K).

No significant changes in sensitivity to F1F0 ATP synthase inhibition were observed in NCI‐H82 cells upon CKMT1 deletion (Figure S2A–D, Supporting Information). To explore if this lack of effect stems from redundant functions between CKB and CKMT1, we generated CKB and CKMT1 double‐null NCI‐H82 cells (Figure S2M, Supporting Information). Cell viability assays revealed that further inhibition of CKMT1 did not affect the sensitivity of NCI‐H82‐shCKB cells to Gboxin or Oligomycin A treatment (Figure S2N,O, Supporting Information). These results thus suggest that CKB may play a dominant role in determining the cell's reliance on F1F0 ATP synthase for survival.

We further investigated the role of CKB in cell sensitivity to the inhibition of other OXPHOS complexes. Intriguingly, cell viability assays showed that CKB knockdown did not confer resistance to Rotenone (a classical complex I inhibitor in ETC) and Antimycin A (a classical complex III inhibitor in ETC) treatment in NCI‐H82 cells (Figure 1L,M). Instead, NCI‐H82 cells lacking CKB displayed increased sensitivity to the inhibition of complex III in OXPHOS by Antimycin A (Figure 1M). These findings highlight the essential role of CKB specifically in determining the cell's reliance on F1F0 ATP synthase and not on other complexes within the OXPHOS machinery.

### 2.3 CKB Deficiency Impairs Mitochondrial ATP Production

**Figure 2:**
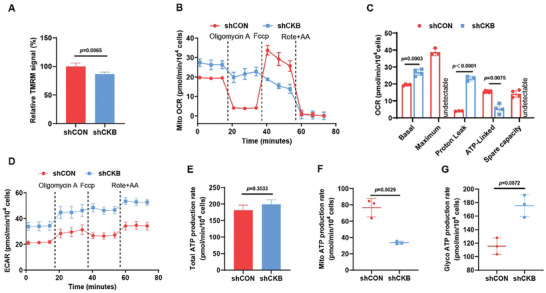
CKB enhances mitochondrial ATP production while suppressing glycolytic ATP generation. A) TMRM staining revealed decreased mitochondrial membrane potential (MMP) in NCI‐H82‐shCKB cells compared to NCI‐H82‐shCON cells. The *p*‐value was calculated using a paired *t*‐test. B,C) Seahorse analyzer assays demonstrated a reduction in ATP‐linked oxygen consumption rate (OCR) and an increase in proton leakage‐induced OCR in NCI‐H82‐shCKB cells compared to NCI‐H82‐shCON cells. Oligomycin A (18 min.), FCCP (36 min.), and a mixture of Rotenone and Antimycin A (Rote+AA, 54 min.) were used. Mean ± SD; *n* = 3. The *p*‐value was calculated using a paired *t*‐test. D) Seahorse analyzer assays indicated enhanced glycolytic activity, as evidenced by an increase in extracellular acidification rate (ECAR) in NCI‐H82‐shCKB cells compared to NCI‐H82‐shCON cells. Oligomycin A (18 min.), FCCP (36 min.), and a mixture of Rotenone and Antimycin A (Rote+AA, 54 min.) were used. Mean ± SD; *n* = 3. The *p*‐value was calculated using a paired *t*‐test. E) XF real‐time ATP rate assay showed no significant difference in total ATP production rate between NCI‐H82‐shCKB and NCI‐H82‐shCON cells. Mean ± SD; *n* = 3. The *p*‐value was calculated using a paired *t*‐test. F,G) XF real‐time ATP rate assay demonstrated reduced mitochondrial ATP production but increased glycolytic ATP production rate in NCI‐H82‐shCKB cells compared to NCI‐H82‐shCON cells. Mean ± SD; *n* = 3. The *p*‐value was calculated using a paired *t*‐test.

The specific and critical role of CKB in determining cancer cell sensitivity to F1F0 ATP synthase inhibition suggests that CKB may influence mitochondrial energy metabolism. While no significant changes in mitochondrial mass were observed in terms of mitochondrial housekeeping proteins (TOM20, VDAC1, and ATP5A1) (Figure S3A,B, Supporting Information), a notable reduction in mitochondrial membrane potential (MMP) was evident in NCI‐H82 cells upon CKB silencing (**Figure** **2**A). This implies disrupted mitochondrial function rather than altered mitogenesis in NCI‐H82‐shCKB cells compared to NCI‐H82‐shCON cells. Consistently, the Agilent Seahorse cell mitochondrial stress test revealed a significant decrease in ATP‐producing‐linked mitochondrial oxygen consumption rates (OCR) but a substantial increase in proton leakage within the OXPHOS machinery in NCI‐H82‐shCKB cells compared to those in NCI‐H82‐shCON cells (Figure 2B,C). It is important to note that the maximum OCR capacity for NCI‐H82 cells with CKB knockdown could not be detected, likely due to the collapse of OXPHOS complexes upon treatment with FCCP (a lipophilic cationic proton translocator) (Figure 2B,C). These findings suggest that CKB is essential for maintaining efficient mitochondrial ATP production in cells sensitive to F1F0 ATP synthase inhibition. In response to the decreased mitochondrial ATP production, glycolysis, as indicated by the extracellular acidification rate (ECAR), was significantly increased in NCI‐H82 cells with CKB silencing (Figure 2D). While there was no significant change in the total ATP production rate between NCI‐H82‐shCON and NCI‐H82‐shCKB cells, the contribution of ATP from mitochondria was markedly reduced in cells lacking CKB compared to control cells (Figure 2E–G; Figure S3C, Supporting Information). Similar to the findings in NCI‐H82‐shCKB cells, the mitochondrial ATP production rate in 786‐O cells, which lack detectable CKB expression, is notably lower compared to that of G‐401 and NCI‐H82 cells (Figure S3D, Supporting Information).

### 2.4 CKB Suppresses Mitochondrial Calcium Levels and mPTP Activity

**Figure 3:**
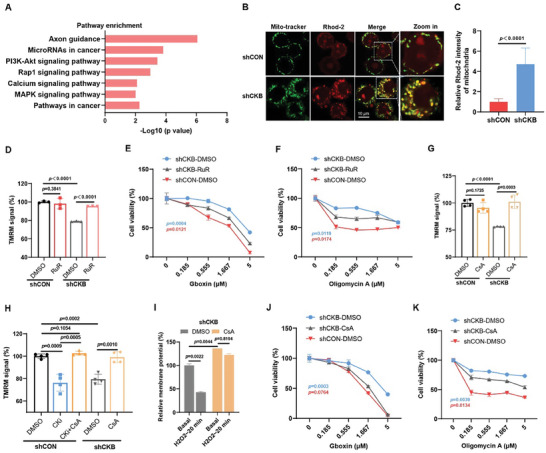
CKB enhances mitochondrial ATP production by inhibiting mCa^2+^ levels and mPTP activity. A) KEGG enrichment analysis was conducted on upregulated genes in NCI‐H82‐shCKB cells compared to NCI‐H82‐shCON cells. B,C) Confocal microscope images display increased mCa^2+^ in NCI‐H82‐shCKB cells compared to NCI‐H82‐shCON cells. Rhod‐2 probe (5 µM) was used to detect mCa^2+^, followed by incubation with 200 nm Mito‐tracker Green. C) quantification of (B). Mean ± SD; *n* = 3. The *p*‐value was calculated using a paired *t*‐test. D) TMRM staining for MMP of NCI‐H82‐shCON and NCI‐H82‐shCKB cells treated with or without Ruthenium red (RuR, 100 µm) for 30 min. Mean ± SD; *n* = 3. The *p*‐value was calculated using a paired *t*‐test. E,F) Cell viability analysis indicates that mCa^2+^ inhibition by RuR sensitizes NCI‐H82‐shCKB cells to the inhibition of F1F0 ATP synthase by Gboxin (E) and Oligomycin A (F). Cells were pretreated with 100 µm RuR for 30 min, followed by incubation at various concentrations of Gboxin and Oligomycin A with or without RuR for 3 days. *n* = 3. The *p*‐value was calculated using a paired *t*‐test. G) mPTP inhibition by Cyclosporin A (CsA, 3 µm, 20 min.) increases the MMP of NCI‐H82‐shCKB cells but not that of NCI‐H82‐shCON cells. MMP was detected by TMRM staining. Mean ± SD; *n* = 4. The *p*‐value was calculated using a paired *t*‐test. H) CKi (3 µm, 6 h) treatment decreases MMP, which is rescued by co‐treatment with CsA (3 µm, 30 min) in NCI‐H82‐shCON cells. MMP was detected by TMRM staining. Mean ± SD; *n* = 4. The *p*‐value was calculated using a paired *t*‐test. I) H_2_O_2_ (0.01%) reduces basal membrane potential in NCI‐H82‐shCKB cells. CsA mPTP inhibition enhances NCI‐H82‐shCKB membrane potential and renders them insensitive to H_2_O_2_. JC‐1 (0.1 µm) staining for MMP was detected after cells were pretreated with or without 3 µm CsA for 30 min. Mean ± SD; *n* = 3. The *p*‐value was calculated using a paired *t*‐test. J,K) Cell viability analysis shows that CsA mPTP inhibition sensitized NCI‐H82‐shCKB cells to the blockage of F1F0 ATP synthase by Gboxin (J) and Oligomycin A (K). Cells were pretreated with CsA for 30 min, followed by incubation at various concentrations of Gboxin and Oligomycin A for 3 days. Mean ± SD; *n* = 3. The *p*‐value was calculated using a paired *t*‐test.

To elucidate the molecular mechanisms governing CKB‐regulated regulation of mitochondrial energy production, we conducted a transcriptome analysis using NCI‐H82‐shCON and NCI‐H82‐shCKB cells. The KEGG pathway analysis of differentially expressed genes (DEGs) unveiled the upregulation of several metabolism‐regulating pathways in NCI‐H82‐shCKB cells in comparison to NCI‐H82‐shCON cells (**Figure** **3**A). These pathways encompass the AKT signaling pathway, calcium signaling pathway, and MAPK signaling pathway (Figure 3A).

Calcium is a key regulator of various mitochondrial functions, including mPTP activity, energy production, and mitochondrial morphology.^[^ ^18^ ^]^ To investigate the potential involvement of CKB in mCa^2+^ regulation, we employed confocal laser scanning microscopy. The results, illustrated in Figure 3B,C, unveiled a notable elevation in mCa^2+^ levels in NCI‐H82‐shCKB cells compared to that in NCI‐H82‐shCON cells. In contrast, overexpress of CKB greatly reduces the mCa^2+^ levels in 786‐O cells (Figure S4A,B, Supporting Information). These data thus indicate a suppressive role of CKB on mCa^2+^ levels. Moreover, the increased mCa^2+^ levels likely contribute to the impaired mitochondrial function in NCI‐H82‐shCKB cells, as treatment with Ruthenium red (RuR), an inhibitor of the mCa^2+^ uniporter (MCU),^[^ ^19^ ^]^ restored the decreased MMP in these cells (Figure 3D; Figure S4C, Supporting Information). It is noteworthy that RuR treatment decreased mCa^2+^ levels without affecting MMP in NCI‐H82‐shCON cells (Figure 3D; Figure S4D, Supporting Information). Additionally, RuR treatment sensitized NCI‐H82‐shCKB cells, but not NCI‐H82‐shCON cells, to F1F0 ATP synthase inhibition (Figure 3E,F; Figure S4E–H, Supporting Information). These results strongly indicate that mCa^2+^ plays a pivotal role in CKB‐regulated mitochondrial ATP production.

Considering the elevated mCa^2+^ levels (Figure 3B,C) and increased proton leakage in OXPHOS machinery (Figure 2B,C), coupled with the reduced MMP in NCI‐H82 cells upon CKB knockdown (Figure 2A), we delved into the potential role of CKB in regulating mPTP activity. Figure 3G demonstrates that there was no discernible change in MMP in NCI‐H82‐shCON cells following CsA treatment, a conventional mPTP inhibitor, indicating minimal to no mPTP activity in NCI‐H82‐shCON cells under standard culture conditions. In contrast, CsA treatment significantly boosted MMP in NCI‐H82‐shCKB cells or NCI‐H82 cells treated with CKB inhibitor CKi‐1 (CKi),^[^ ^20^ ^]^ implying substantial basal mPTP activity upon CKB was inhibited in NCI‐H82 cells (Figure 3G,H; Figure S4I, Supporting Information). This active mPTP in NCI‐H82‐shCKB cells was further corroborated when these cells were exposed to H_2_O_2_, a well‐known mPTP activator. As shown in Figure 3I, H_2_O_2_ treatment led to a notable MMP decrease in NCI‐H82‐shCKB cells, which could be rescued by preincubation with the mPTP inhibitor CsA. Further, in line with the inhibitory role of CKB in mPTP activation, overexpression of CKB reduces mPTP activity in 786‐O cells (Figure S4J, Supporting Information).

We then investigated whether the heightened mPTP activity contributes to reduced reliance of cells on F1F0 ATP synthase. As depicted in Figure 3J,K, Figure S4K,L (Supporting Information) and Figure S4H (Supporting Information), while CsA treatment did not impact the sensitivity of NCI‐H82‐shCON cells to the inhibition of F1F0 ATP synthase by Gboxin and Oligomycin A, CsA treatment markedly sensitizes NCI‐H82‐shCKB cells to the inhibition of F1F0 ATP synthase reflected by decreased IC50s and increased mitochondrial stress responses. These observations strongly suggest that increased mPTP activity plays a crucial role in the diminished mitochondrial ATP production in NCI‐H82 cells following CKB protein knockdown.

### 2.5 CKB and Its Product Phosphocreatine Suppress AKT Activation

**Figure 4:**
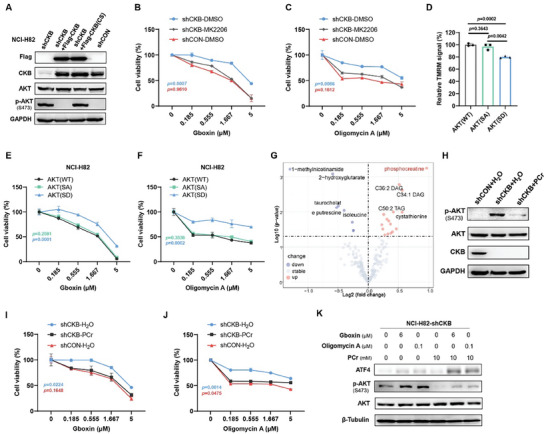
CKB and phosphocreatine sensitize cells to F1F0 ATP synthase inhibition by suppressing AKT activation. A) Western blot analysis demonstrates that expression of Flag‐CKB but not Flag‐CKB(CS) suppresses AKT activation in NCI‐H82‐shCKB cells. *n* = 3. B,C) Cell viability analysis demonstrates that AKT inactivation sensitizes NCI‐H82‐shCKB cells to Gboxin and Oligomycin A treatments. Cells were treated with a series of concentrations of Gboxin (B) and Oligomycin A (C) with or without a 60‐min pretreatment of MK2206 (AKT inhibitor, 5 µm), followed by cell viability analysis 3 days later. Mean ± SD; *n* = 3. The *p*‐value was calculated using a paired *t*‐test. D) TMRM staining shows that expression of a phosphorylation‐mimicking mutant AKT (AKT(SD)) but not wildtype AKT (AKT(WT)) or a phosphorylation‐resistant mutant AKT (AKT(SA)) decreases mitochondrial membrane potential. Mean ± SD; *n* = 3. The *p*‐value was calculated using a paired *t*‐test. E,F) Cell viability analysis demonstrates that expression of AKT(SD) but not wildtype or AKT(SA) mutant renders NCI‐H82 cells resistant to F1F0 ATP synthase inhibition by Gboxin (E) or Oligomycin A (F). Cells were treated with various concentrations of Gboxin or Oligomycin A as indicated for 3 days before cell viability analysis. Mean ± SD; *n* = 3. The *p*‐value was calculated using a paired *t*‐test. G) Metabolite analysis reveals a high level of phosphocreatine (PCr) in Oligomycin A‐sensitive cells compared to Oligomycin A‐resistant cells. Cell metabolomics data are from CCLE. H) Western blot analyses demonstrate that phosphocreatine treatment reduces AKT activation in NCI‐H82‐shCKB cells. Cells were treated with or without 10 mm PCr for 4 h. I,J) Cell viability analysis shows that PCr treatment sensitizes NCI‐H82‐shCKB cells to F1F0 ATP synthase inhibition by Gboxin and Oligomycin A. NCI‐H82‐shCKB cells were incubated with various concentrations of Gboxin or Oligomycin A with or without 10 mm PCr pretreatment for 60 min, followed by cell viability analysis after 3 days. Mean ± SD; *n* = 3. The *p*‐value was calculated using a paired *t*‐test. K) Western blot analysis for ATF4 expression demonstrates treatment of PCr sensitized NCI‐H82‐shCKB cells to the inhibition of F1F0 ATP synthase by Gboxin and Oligomycin A. Cells were pretreated with or without 10 mm PCr for 1 h, followed by incubation with Gboxin and Oligomycin A for another 3 h. *n* = 3.

Transcriptome analysis also revealed an upregulation of AKT signaling target genes in NCI‐H82 cells upon CKB silencing (Figure 3A). Correspondingly, robust AKT activation was observed in NCI‐H82‐shCKB cells compared to NCI‐H82‐shCON cells, as shown by western blot analysis (**Figure** **4**A). This AKT activation in NCI‐H82‐shCKB cells is specifically attributed to the absence of CKB, as re‐expression of CKB reversed this phenotype (Figure 4A). On the other hand, overexpression of CKB significantly inhibits AKT activation in 786‐O cells (Figure S5A, Supporting Information). Furthermore, the inhibition of AKT by MK2206 (a selective allosteric inhibitor of AKT)^[^ ^21^ ^]^ significantly enhanced the sensitivity of NCI‐H82‐shCKB cells to the inhibition of F1F0 ATP synthase by Gboxin or Oligomycin A treatment (Figure 4B,C; Figure S5B,C, Supporting Information), whereas MK2206 treatment had no impact on the sensitivity of NCI‐H82‐shCON cells to F1F0 ATP synthase blockage (Figure S5D,E, Supporting Information). These findings indicate that AKT signaling plays a pivotal role in the CKB‐mediated regulation of mitochondrial ATP production.

AKT activation requires phosphorylation. To further investigate the involvement of AKT in CKB‐regulated mitochondrial energy metabolism, we introduced wild‐type AKT (AKT(WT)), AKT S473A mutation (AKT(SA), which is resistant to phosphorylation), and AKT S473D mutation (AKT(SD), a phosphorylation‐mimicking variant) into NCI‐H82 cells (Figure S5F, Supporting Information).^[^ ^22^ ^]^ As shown in Figure 4D–F, the expression of AKT(SD), but not AKT(SA) or AKT(WT), led to a decrease in MMP and conferred resistance to the inhibition of F1F0 ATP synthase. These results, observed in NCI‐H82 cells expressing the phosphorylation‐mimicking mutant (AKT(SD)), mirrored the findings in CKB‐deficient NCI‐H82 cells, strongly indicating that AKT activation is involved in the impaired mitochondrial ATP production in NCI‐H82‐shCKB cells.

CKB facilitates the conversion of creatine to phosphocreatine in cells.^[^ ^1a^ ^]^ Metabolomics data reveal differential levels of various metabolites between cells that are susceptible and resistant to F1F0 ATP synthase inhibition. Notably, phosphocreatine is the most abundantly accumulated metabolite in susceptible cells (Figure 4G). To explore the potential involvement of phosphocreatine in CKB‐mediated AKT signaling and mitochondrial ATP production, we treated NCI‐H82‐shCON and NCI‐H82‐shCKB cells with phosphocreatine. As shown in Figure 4H–K, phosphocreatine treatment not only reversed the heightened AKT phosphorylation in CKB‐deficient cells but also increased the sensitivity of NCI‐H82‐shCKB cells to F1F0 ATP synthase inhibition. Conversely, phosphocreatine treatment did not affect the sensitivity of NCI‐H82‐shCON cells to F1F0 ATP synthase inhibition (Figure S5G,H, Supporting Information). In addition, unlike the expression of Flag‐tagged wildtype CKB (Flag‐CKB) protein, the expression of the enzymatic inactive CKB mutant Flag‐CKB(C283S,CS),^[^ ^23^ ^]^ which is unable to produce phosphocreatine from creatine, is unable to suppress AKT activation (Figure 4A). These findings collectively indicate that phosphocreatine, a CKB‐derived product, plays a crucial role in modulating cellular energy metabolism in cells dependent on F1F0 ATP synthase for viability.

### 2.6 CKB Suppresses mCa^2+^ and mPTP Activity by Inhibiting AKT Function

**Figure 5:**
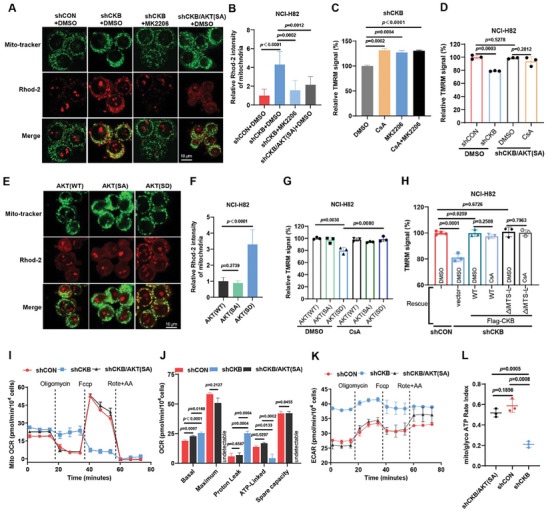
CKB enhances mitochondrial ATP production by inhibiting AKT activation. A,B) Representative confocal images demonstrate that AKT inhibition by MK2206 treatment or expression of a phosphorylation‐resistant mutant (AKT(SA)) reduces mitochondrial calcium (mCa^2+^) levels in NCI‐H82‐shCKB cells. Cells were incubated with 5 µm Rhod‐2 probe for 30 min to assess mCa^2+^ levels, and mitochondria were stained with MitoTracker Green. Cells were treated with or without 5 µm MK2206 for 1 h. *n* = 3. B) Quantification of (A). Mean ± SD; *n* = 3. The *p*‐value was calculated using a paired *t*‐test. C) TMRM staining for mitochondrial membrane potential (MMP) in NCI‐H82‐shCKB cells treated with the mitochondrial permeability transition pore (mPTP) inhibitor Cyclosporin A (CsA, 3 µm, 30 min), AKT inhibitor MK2206 (5 µm, 30 min), or a combination of CsA and MK2206 for 30 min. Mean ± SD; *n* = 3. The *p*‐value was calculated using a paired *t*‐test. D) TMRM staining for MMP in cells as indicated treated with DMSO or mPTP inhibitor CsA shows that expression of AKT(SA) rescues decreased MMP in NCI‐H82‐shCKB cells, and CsA treatment does not further increase MMP in NCI‐H82‐shCKB cells expressing AKT(SA). CsA, 3 µm pretreated for 30 min. Mean ± SD; *n* = 3. The *p*‐value was calculated using a paired *t*‐test. E,F) Representative confocal images reveal increased mCa^2+^ in NCI‐82 cells expressing AKT(SD) but not AKT(SA) or AKT(WT). Rhod‐2 (5 µm, 30 min) staining for mCa^2+^ levels and MitoTracker Green (200 nm, 20 min) for mitochondria. *n* = 3; F) quantification of (E). Mean ± SD; *n* = 3. The *p*‐value was calculated using a paired *t*‐test. G) TMRM staining of cells as indicated shows decreased MMP in cells transfected with AKT(SD) but not those transfected with AKT(WT) or AKT(SA). Treatment with the mPTP inhibitor CsA rescues decreased MMP in cells transfected with AKT(SD). CsA 3 µm, 20 min. Mean ± SD; *n* = 3. The *p*‐value was calculated using a paired *t*‐test. H) Expression of Flag‐CKB(WT) or Flag‐CKB(ΔiMTS‐L) increases the MMP in NCI‐H82‐shCKB cells. Treatment of CsA (3 µm, 30 min.) does not affect MMP in NCI‐H82‐shCKB cells expressing Flag‐CKB(WT) or Flag‐CKB(ΔiMTS‐L). *n* = 3 or 4. The *p*‐value was calculated using a paired *t*‐test. I,J) Seahorse analyzer assays demonstrate that expression of AKT(SA) rescues decreased ATP‐linked oxygen consumption rate (OCR) and increased proton leakage‐induced OCR in NCI‐H82‐shCKB cells. Cells were treated with Oligomycin A (18 min), FCCP (36 min), and a mixture of Rotenone and Antimycin A (Rote + AA, 54 min). Mean ± SD; *n* = 3. J) Quantification of (I). Mean ± SD; *n* = 3. The *p*‐value was calculated using a paired *t*‐test. K) Seahorse analyzer assays reveal that expression of AKT(SA) rescues enhanced glycolytic activity in NCI‐H82‐CKB cells. Cells were treated with Oligomycin A (18 min), FCCP (36 min), and a mixture of Rotenone and Antimycin A (Rote + AA, 54 min). Mean ± SD; *n* = 3. The *p*‐value was calculated using a paired *t*‐test. L) XF real‐time ATP rate assay shows expression of AKT(SA) in NCI‐H82‐shCKB cells increases mitochondrial (mito)/glycolytic (glyco) ATP production rate compared to that in control cells. Mean ± SD; *n* = 3. The *p*‐value was calculated by paired *t*‐test.

Our results indicate that silencing CKB in NCI‐H82 cells elevates mCa^2+^ levels and mPTP activity (Figure 3). Subsequently, we explored the role of AKT in the increased mCa^2+^ levels and mPTP activity resulting from CKB silencing in NCI‐H82 cells. As shown in **Figure** **5**A,B, inhibition of AKT using the AKT inhibitor MK2206 or expression of the inactive version of AKT (AKT(SA)) (Figure S6A, Supporting Information) reduced mCa^2+^ levels in CKB‐silenced NCI‐H82 cells to levels comparable to those in NCI‐H82‐shCON cells. Moreover, AKT activation appears crucial for mPTP opening in NCI‐H82‐shCKB cells, as treatment with MK2206 or expression of AKT(SA) restored the MMP to levels akin to those observed with the mPTP‐specific inhibitor, CsA (Figure 5C,D), while MK2206 treatment had no impact on MMP levels in NCI‐H82‐shCON cells (Figure S6B, Supporting Information). Notably, simultaneous blockade of AKT activation and mPTP opening in NCI‐H82‐shCKB cells did not lead to further MMP elevation (Figure 5C,D), strongly indicating that AKT activation is responsible for the increased mCa^2+^ levels and mPTP activity triggered by CKB silencing in NCI‐H82 cells. This inference is reinforced by our experiments involving the expression of AKT mutants and wild‐type AKT in NCI‐H82 cells. As illustrated in Figure 5E–G, expression of the phosphorylation‐mimicking AKT mutant (AKT(SD)), but not the AKT inactivation mutant (AKT(SA)) or wildtype AKT (AKT(WT)), was adequate to raise mCa^2+^ levels and mPTP activity in NCI‐H82 cells.

It has been previously documented that CKB contains an internal mitochondrial‐targeting signal‐like (iMTS‐L) sequence conferring mitochondrial localization.^[^ ^24^ ^]^ In order to explore whether CKB's regulation of mitochondrial ATP production depends on its mitochondrial localization, we introduced Flag‐CKB(ΔiMTS‐L) and Flag‐CKB into NCI‐H82‐shCKB cells. As depicted in Figure S6C,D (Supporting Information), despite losing its mitochondrial localization, Flag‐CKB(ΔiMTS‐L) is able to rescue the decreased MMP level and increased mPTP activity in NCI‐H82‐shCKB cells, similar to the effect observed with Flag‐CKB (Figure 5H). This suggests that cytosolic CKB may play a crucial role in the regulation of mPTP activity and mitochondrial ATP production.

### 2.7 CKB Enhances Mitochondrial ATP Production by Inhibiting AKT Activity

We then investigated the potential role of AKT signaling in CKB‐mediated regulation of mitochondrial ATP production. As shown in Figure S6E,F (Supporting Information), inhibition of AKT activity by expression AKT(SA) in CKB deficient cells significantly increased the sensitivity to the inhibition of F1F0 ATP synthase by Gboxin and Oligomycin A. Furthermore, AKT inhibition through the expression of AKT(SA) also rescued CKB knockdown‐induced proton leakage, decreased ATP‐linked OCR, and increased glycolysis (Figure 5I–L). Notably, while we could not detect maximum OCR in NCI‐H82‐shCKB cells, similar levels of maximum OCR were observed in AKT(SA) expressing NCI‐H82‐shCKB cells compared to those in NCI‐H82‐shCON cells (Figure 5J). These findings strongly suggest that AKT suppression by CKB is crucial for maintaining efficient mitochondrial ATP production in NCI‐H82 cells. Consistent with this, NCI‐H82 cells expressing the phosphorylation‐mimicking AKT mutant (AKT(SD)) showed increased proton leakage, reduced mitochondrial ATP production, and elevated glycolytic ATP production, similar to the observations in NCI‐H82‐shCKB cells (Figure S6G–J, Supporting Information).

### 2.8 CKB‐AKT Signaling Modulates Mitochondrial ATP Production In Vivo

**Figure 6:**
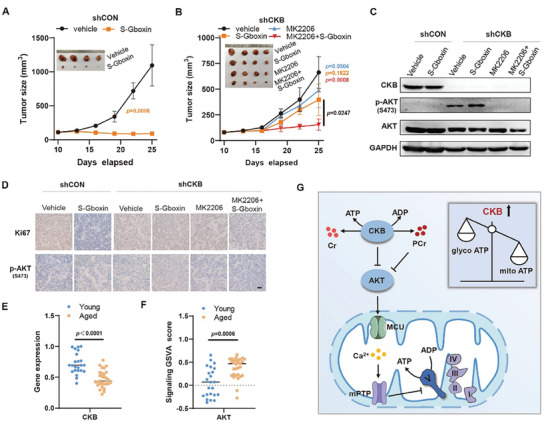
The CKB‐AKT signaling pathway enhances mitochondrial ATP production in vivo. A,B) NCI‐H82‐CKB tumors exhibit resistance to S‐Gboxin treatment compared to NCI‐H82‐shCON tumors. Treatment with the AKT inhibitor MK2206 sensitizes NCI‐H82‐CKB tumors to S‐Gboxin treatment. S‐Gboxin, a stable analog of Gboxin suitable for in vivo administration. NCI‐H82‐shCON (A) and NCI‐H82‐CKB cells (B) were subcutaneously injected into the flanks of nude mice. Tumor‐bearing mice were treated with vehicle or S‐Gboxin (10 mg kg^−1^; intraperitoneally) and/or MK2206 (12.5 mg kg^−1^; intraperitoneally) daily for 14 days as indicated. Tumor volume was measured every 3 days. Mean ± SD; *n* = 5. The *p*‐value was calculated using a paired *t*‐test. C) Molecular analysis of p‐AKT and CKB expression in tumors from (A and B). *n* = 3. D) Representative images of H&E staining and IHC analysis for p‐AKT and Ki67 expression in tumors from (A and B). *n* = 3; scale bar = 50 µm. E,F) Reduced transcription of CKB (E) and upregulated transcription of AKT signaling‐related genes (F) are observed in peripheral blood mononuclear cells (PBMCs) from the Aged group compared to those in the Young group. PBMC samples from 23 healthy individuals aged 20–29 (Young group) and thirty healthy individuals aged 50–59 (Aged group) were collected and sequenced for whole‐genome transcription. Gene Set Variation Analysis (GSVA) is used to evaluate the transcription level of AKT signaling‐related genes in the Young and Aged groups. Mean ± SD; The *p*‐value was calculated using a paired *t*‐test. G) Model for CKB‐regulated signaling cascade boosting mitochondrial ATP production. CKB and its product phosphocreatine (PCr) enhance mitochondrial energy production by inhibiting AKT activation, which subsequently suppresses mCa^2+^ levels and prevents mPTP activity, ensuring high efficiency of OXPHOS in producing mitochondrial ATP while suppressing glycolytic ATP production.

Our previous results indicated the sensitivity of certain cancer types to OXPHOS inhibitors.^[^ ^15b^ ^]^ This was validated using NCI‐H82‐shCON cells, where treatment with S‐Gboxin, an inhibitor of F1F0 ATP synthase used in preclinical studies,^[^ ^15a^ ^]^ significantly decreased the growth of subcutaneously transplanted tumors by NCI‐H82‐shCON cells (**Figure** **6**A). Consistent with the reduced sensitivity of NCI‐H82‐shCKB cells to F1F0 ATP synthase inhibition, treatment with S‐Gboxin had a notably diminished effect on the growth of NCI‐H82‐shCKB tumors compared to NCI‐H82‐shCON tumors (Figure 6B). Our cell culture data demonstrated that AKT inactivation sensitizes NCI‐H82‐shCKB cells to F1F0 ATP synthase inhibition. Therefore, we explored the combined treatments of AKT activation inhibitors and F1F0 ATP synthase inhibitors. As shown in Figure 6B–D, while treatment with the AKT inhibitor (MK2206), which significantly inhibits AKT activation in the transplanted NCI‐H82‐shCKB tumors, did not significantly impact the growth of NCI‐H82‐shCKB tumors alone, the combined treatments of S‐Gboxin and MK2206 showed a synergistic inhibitory effect on NCI‐H82‐shCKB tumor growth. Moreover, the reduced tumor growth observed with the various treatments correlated with decreased Ki67‐positive cells (a proliferation marker for cancer cells) (Figure 6D; Figure S7, Supporting Information). These results collectively suggest that CKB‐mediated AKT suppression may play a critical role in maintaining mitochondrial energy production and tumor sensitivity to F1F0 ATP synthase inhibition in vivo.

Upregulated AKT activation and enhanced mPTP activity have been observed in aged tissues and cells,^[^ ^10^, ^25^ ^]^ prompting us to explore the potential implications of our study findings on the aging process. Peripheral blood mononuclear cells (PBMCs) were obtained from 23 healthy individuals aged 20–29 (Young group) and thirty sex‐matched healthy individuals aged 50–59 (Aged group) whole genome transcription sequencing. As depicted in Figure 6E,F, the expression of CKB was found to be reduced in the aged group compared to the young group. In line with the inhibitory function of CKB on AKT activation, genes downstream of AKT signaling pathway were upregulated in the aged group. These data suggest that the diminished inhibition of AKT activity by CKB may contribute to the increased mPTP activity and reduced efficiency in mitochondrial ATP production observed in aged cells.^[^ ^4c^ ^]^

## 3 Discussion

CKs are recognized for their pivotal role in reversible phosphate group transfer between ATP and creatine, acting as a cellular energy reservoir.^[^ ^1^ ^]^ However, their contribution to ATP generation has remained ambiguous. In our ongoing exploration of cellular responses to F1F0 ATP synthase inhibition by Gboxin, we have noted a robust positive correlation between CK expression and cellular sensitivity to F1F0 ATP synthase inhibition. Our data also indicate that it is CKB and likely not other CK isoforms, that plays a crucial role in determining cellular dependence on mitochondrial ATP production. CKB accomplishes this by impeding AKT activation, thereby reducing mCa^2+^ levels and curtailing mPTP activity, ensuring the efficiency of OXPHOS in generating mitochondrial ATP (Figure 6G). Additionally, our discoveries suggest that CKB's regulation of mitochondrial ATP production is probably conserved in specific types of cancer cells thriving in a murine tumor model and diminishes with aging. These outcomes signify a groundbreaking revelation: CK proteins, traditionally associated with energy buffering, govern mitochondrial ATP production in both in vitro and in vivo cell environments.

The ETC, comprising complexes I–IV, collaborates with the F1F0 ATP synthase in OXPHOS to generate ATP.^[^ ^6^ ^]^ Apart from ATP synthesis, research indicates that the ETC also participates in various processes such as biosynthesis.^[^ ^26^ ^]^ Our findings reveal that knocking down CKB confers resistance to inhibitors of the F1F0 ATP synthase while not affecting inhibitors of complex I and complex III, suggesting a specific regulation of CKB on the F1F0 ATP synthase, the site of mitochondrial ATP production. This phenomenon may be elucidated by the heightened mPTP activity observed in CKB‐deficient NCI‐H82 cells, as increased mPTP opening disrupts the coordination between the ETC and F1F0 ATP synthase by elevating proton leakage. Nevertheless, numerous unresolved queries warrant further exploration. One such inquiry pertains to why CKB‐deficient NCI‐H82 cells exhibit heightened reliance on glycolysis for ATP production (Figure 2B–G; Figure S3C, Supporting Information), yet display increased sensitivity to complex III inhibition in OXPHOS compared to wildtype NCI‐H82 cells (Figure 1M).

The AKT signaling pathway has been implicated in governing various mitochondrial functions, impacting processes such as mCa^2+^ levels, mitochondrial metabolism, and mitogenesis.^[^ ^9^ ^]^ Our investigation unveiled that heightened AKT activity in CKB‐deficient NCI‐H82 cells leads to elevated mCa^2+^ levels without affecting mitogenesis. Additionally, reducing mCa^2+^ levels mimics the effects of AKT inhibition in CKB‐deficient NCI‐H82 cells (Figures 3D–F and 4B,C), strongly indicating that AKT‐induced alterations in mCa^2+^ are pivotal for CKB‐mediated regulation of mitochondrial ATP production. Although the precise mechanism through which CKB suppresses AKT activation remains elusive in our current study, our data suggest that phosphocreatine, a product of CKB activity, may play a critical role in this regulatory process.

Mitochondrial dysfunction stands as a prominent feature of both cancer and aging.^[^ ^27^ ^]^ Our research has pinpointed a subset of cancer cells exhibiting high expression of CK genes, rendering them vulnerable to inhibitors of mitochondrial ATP production both in vitro and in vivo. With the emergence of various agents targeting mitochondrial energy in the context of tumor therapy in preclinical and clinical settings,^[^ ^28^ ^]^ our study not only enhances our comprehension of mitochondrial reprogramming in cancer cells but also lays the groundwork for the identification of biomarkers for these antitumor agents. Conversely, aged tissues and cells exhibit heightened mPTP activity and inefficient ATP generation.^[^ ^25^ ^]^ Our study demonstrates a decline in CKB expression in PBMCs from aged individuals, accompanied by an increase in the expression of genes targeted by the AKT signaling pathway in these cells. This suggests a diminished functionality of the CKB‐AKT‐F1F0 ATP synthase signaling cascade in aged individuals, warranting further exploration.

## 4 Experimental Section

### Cell Lines and Cell Culture Conditions

NCI‐H82, G‐401, MDA‐MB‐453, 786‐O, CFPAC‐1 cells, and HEK293T were obtained from ATCC, SF126 cell was obtained from JCRB Cell Bank. NCI‐H82, G‐401, and 786‐O were cultured in RPMI 1640 medium (WISENT, 350‐000‐CL), SF‐126, and HEK293T were cultured in DMEM medium (WISENT, 319‐005‐CL), CFPAC‐1 were cultured in IMDM medium (MINGZHOUBIO, MPM150510), MDA‐MB‐453 was cultured in L‐15 medium (WISENT, 323‐050‐CL), each medium supplemented with 10% fetal bovine serum, 1% penicillin/streptomycin. MDA‐MB‐453 was maintained in 100% air at 37 °C and other cells were in 5% CO_2_ at 37 °C.

### Lentiviral Transduction and Generation of Stable Cell Lines

The CKB knockdown sequence was subcloned into PLVX‐U6‐sh4‐CMV and single‐guide RNA (sgRNAs) targeting CKMT1 was subcloned into LentiCRISPRv2. The full length of cDNA encoding human CKB was generated from NCI‐H82 cell mRNA and was subcloned into pLVX‐pCMV‐pPGK.

Lentiviruses were produced by transfecting plasmids together with helper plasmids PSPAX2 and PMD.2 g into HEK293T cells at the ratio of 6:3:1. Cell supernatants were collected 48 h after transfection, to obtain stable cell lines, cells were infected for 48 h with lentiviral supernatants diluted 1:1 with normal culture media in the presence of 5 µg mL^−1^ of Polybrene. Cells were subjected to Puromycin selection after infection.

Lentiviral shRNAs, sgRNA, and other primers were obtained from Genewiz. Sequences used for targeting shRNAs is as follows:

CKB‐1: CCCTGCTGCTTCCTAACTTAT
CKB‐2: GTCTAAGGACTATGAGTTCAT

Sequences used for targeting sgRNA are as follows:

CKMT1‐1: GAGCGGGGTCTCTAGCCGCT
CKMT1‐2: GACGGAGGCTGTATCCCCCG

### DNA Construction and Mutagenesis

PCR‐amplified Flag‐CKB or HA‐AKT1 was cloned in pLVX‐pCMV‐pPGK. Primers are:

Flag‐CKB‐Fw:

TCGAGCTCAAGCTTCGAATTCATGGACTACAAAGACGATGACGACAAGATGCCCTTCTCCAACAGCCA,

Flag‐CKB‐Rev: TTATCTAGAGTCGCGGGATCCTCATTTCTGGGCAGGCATGA.

HA‐AKT1‐Fw: TCGAGCTCAAGCTTCGAATTCATGTACCCTTATGACGTACCTGACTATGCTGGAATGAGCGACGTGGCTATTGTG

HA‐AKT1‐Rev: ttatctagagtcgcgggatccTCAGGCCGTGCCGCTGGC

The following mutations were generated using a QuickMutation Site‐directed Gene Mutagenesis Kit: QuickMutation.

Primers are:

AKT1‐S473A‐Fw:

CACTTCCCCCAGTTCGCCTACTCGGCCAGCGGC

AKT1‐S473A‐Rev:

GCCGCTGGCCGAGTAGGCGAACTGGGGGAAGTG

AKT1‐S473D‐Fw:

CACTTCCCCCAGTTCGACTACTCGGCCAGCGGC

AKT1‐S473D‐Rev:

gccgctggccgagtaGTCgaactgggggaagtg

CKB‐C283S‐FW:

ctgggctacatcctcaccagcccatccaacctgggcac

CKB‐C283S‐Rev: gtgcccaggttggatgggctggtgaggatgtagcccaG

CKB‐ΔiMTS‐L‐FW:

TACGTGCTGAGCTCGGAGGTGGAGACGGGCGAGAGCATCGAGGGCTTC TGCCTCCCC

CKB‐ΔiMTS‐L‐Rev:

GGGGAGGCAGAAGCCCTCGATGCTCTCGCCCGTCTCCACCTCCGAGCTCAGCACGTAG

### Western Blot

Cells were washed with ice‐cold phosphate‐buffered saline (PBS), and total protein was extracted by using a Whole Cell Lysis Assay (KGP2100, KeyGEN BioTECH). After centrifugation at 12 000 g for 5 min, the supernatants were collected and protein concentrations were determined using the BCA Protein Quantification Kit (23225, Yeasen, 20201ES) with bovine serum albumin as a protein standard. Proteins were separated by 8–10% SDS‐PAGE and then transferred to a 0.45 µm NC membrane.

Then incubated overnight with primary antibodies, the revelation was assessed by specific HRP conjugated secondary antibodies (Proteintech), followed by detection by chemiluminescence (ChemiScope 6200, CLINX).

Anti‐ATF4 (sc‐390063, 1:500) antibody was obtained from Santa Cruz, anti‐CKB antibody (A12631, 1:500) was purchased from ABclonal; Antibodies against CKMT1 (15346‐1‐AP, 1:1000), GAPDH (60004‐1‐Ig, 1:1000), β‐Actin (20536‐1‐AP, 1:1000), HA‐tag (51064‐2‐AP, 1:1000) and Flag‐tag (20543‐1‐AP, 1:1000) were obtained from Proteintech; Antibodies against pS6 (4856S, 1:1000), S6 (2217S, 1:1000), β‐Tubulin (2146S, 1:1000), p‐AKT (9271S, 1:1000) and AKT (9272S, 1:1000) were purchased from Cell Signaling Technology.

### Cell Viability Detection: ATP assay

Cell viability was detected using Cell Titer Glo (Promega) ATP assay, in brief, 500–1500 cells per well were seeds in a 96‐well plate, after incubated overnight then treated with different reagents for 3 days. Following equilibrating the plate at room temperature, add CellTiter‐Glo Reagent, and mix content to induce cell lysis, after 3 min incubation Record luminescence by Microplate Rea der (Tristar^2^ S LB 942, Berthold).

### Mitochondrial Membrane Potential Measurements and mPTP Activity Test

Mitochondrial membrane potential was measured by loading cells with 200 nm tetramethyl rhodamine methyl ester (TMRM, Invitrogen T668) for 30 min under cell culture conditions. Successively, cells were washed twice with cold PBS, then samples were analyzed by flow cytometry (BD LSRFortessa). mPTP Activity test by the opening of mPTP at basal culture condition or in the presence of H_2_O_2_ (0.01%), and cells were treated with 5,5′,6,6′‐tetrachloro‐1,1′,3,3′‐tetraethylbenzimidazolylcarbocyanine iodide (JC‐1, 0.1 µm, J8030, Solarbio), successively, cells were washed twice with cold PBS, then samples were analyzed by Microplate Rea der (Tristar^2^ S LB 942, Berthold).

### RNA Isolation and Quantitative Real‐Time PCR

Total RNA extraction was performed by RNA isolation Total RNA Extraction Reagent (R401‐01, Vazyme), and reverse transcription assays were performed by PrimeScript RT reagent Kit with gDNA Eraser (#RR047A, TaKaRa) in Thermal Cycler (T100, BioRAD) according to manufacturer's instructions. Real‐time quantitative PCR assays were performed with ChamQ Universal SYBR qPCR Master Mix (Q711‐02, Vazyme) in a Real‐Time PCR System (CFX Connect, BioRAD).

The primers sequences of Homo Sapiens were provided as follows:

GAPDH‐Fw: 5′‐ACAACTTTGGTATCGTGGAAGG‐3′;

GAPDH‐Rev: 5′‐GCCATCACGCCACAGTTTC‐3′;

CKMT1‐Fw: 5′‐AGACCTGTGCCTGCCTGTTC −3′;

CKMT1‐Rev: 5′‐ GGAGGAGAAGGTAAAGTGAGGTGAA‐3′;

CKB‐Fw: 5′‐GCTGCGACTTCAGAAGCGA‐3′;

CKB‐Rev: 5′‐GGCATGAGGTCGTCGATGG‐3′;

### Extracellular Flux Analysis

Oxygen consumption rates (OCR) and extracellular acidification rates (ECAR) were analyzed by Seahorse Flux Analyzer XF96 (Agilent) according to the manufacturer's instructions. XF assay medium‐modified DMEM in the presence of 1 mm pyruvate, 10 mm glucose, and 2 mm glutamine without serum was used for OCR and Real‐Time ATP Rate Assay. On the day following 80–90% confluent cells seeding, cells were equilibrated for 1 h in a 37 °C incubator lacking CO_2_. XF assay medium‐modified DMEM in the presence of 2 mm glutamine without serum was used for the ECAR experiment. The concentration of compounds used was: Oligomycin A (1 µm); Fccp (1 µm); a mixture of rotenone (500 nm) and antimycin A (500 nm); OCR or ECAR data were measured under the condition of the picture described. The XF ATP rate was calculated by the XF‐real‐time‐ATP‐rate‐assay‐report‐generator.

### Calcium Concentration

Measurements of resting mitochondrial Ca^2+^ concentrations were made using cells incubated with 5 µm Rhod‐2 Am probe (Abcam, ab142780) for 30 min to detect mCa^2+^, followed by incubation of 200 nm Mito‐tracker Green for another 20 min before washing and confocal imaging (excitation at 535 nm, emission at >560 nm; Nikon CLSM) or analyzed by Microplate Rea der (Tristar^2^ S LB 942, Berthold).

### Xenograft Mouse Model

All mouse experiments were performed in accordance with protocols approved by the Institutional Animal Care and Use Committees (IACUC) at Tongji University with approved ID number SHDSYY‐2022‐P0032. 5‐weeks old BALB/c nude female mice were bought from Shanghai SLAC Laboratory Animal Company. 3 × 106 cells were then subcutaneously injected into the flanks of nude mice at a volume of 100 µL. After 10 days, mice were treated with S‐Gboxin (5 mg kg^−1^ d^−1^) or MK2206 (12.5 mg kg^−1^ d^−1^) or combined with S‐Gboxin and MK2206 by intraperitoneal injection. Tumor volume was calculated using the following formula, tumor volume = 0.5L × W^2^ (L: tumor length, W: tumor width).

### Immunohistochemistry (IHC) Staining

Tumors were fixed in 4% paraformaldehyde for 24 h, and then paraffined‐embedded tumor sections were dewaxed and rehydrated in gradient‐diluted alcohol. Further incubated with anti‐ki67 antibody (1:100 dilution; CST, 12202) and anti‐p‐AKT antibodies (1:100 dilution; CST, 3787) overnight at 4 ◦C. After washing, incubated with biotinylated secondary antibodies (diluted 1:1000) at 37 ◦C for 1 h. Followed by slide staining with hematoxylin and sealed. Images were captured by Olympus microscopy.

### Gene Expression Analysis

The analysis of CKB and AKT signaling gene expression in the young group (20–30 years) and the aged group (50–59 years) was performed by using the previous PBMC RNA‐seq data (the CNGB Sequence Archive (CNSA) of the China National GeneBank Database (CNGBdb) with accession numbers (CNP0002176).

### Statistical Analysis

For high‐throughput sequencing, gene expression values were log2 transformed or rescaled normalized to range from 0 to 10. Data analysis was performed by GraphPad Prism or R Studio. All statistical tests were comprehensively and clearly illustrated in the parallel figure legends. Two‐tailed unpaired *t*‐test or Wilcox test was used for the comparison between the two groups. Mann–Whitney test was used for the comparison between groups of more than two. Pearson test and Cochran‐Armitage trend test were used for correlation analysis. For comparisons, *p* < 0.05 was presented as statistical significance.

All other data were represented as mean ± SD. Statistical analysis was performed using one or two‐way ANOVA, Mann–Whitney test, or paired Student's *t*‐test. All calculations were performed using GraphPad Prism 8 (GraphPad Software, CA, USA).

## Conflict of Interest

Y.S. holds a share of Nanjing Shijiang Medicine Technology Co. LTD, which develops mitochondrial targeting reagents for human health.

## Author Contributions

Y.F.S. supervised the research. Y.F.S., L.H., H.L.L., H.L. S.J.L., H.L., and J.C. designed the experiments. L.H. conducted the experiments. J.H.L. performed and analyzed bioinformatic analysis. Y.F.S. and L.H. wrote the paper. H.L., H.L.L., S.J.L., Q.X.Y., X.Y.W., and J.C. provided constructive advice and commented on the manuscript.

## Supporting information

Supporting Information

Supplemental Table 1

## Data Availability

The data that support the findings of this study are available from the corresponding author upon reasonable request.
