# Supplementary material for: CKB Promotes Mitochondrial ATP Production by Suppressing Permeability Transition Pore
Source: Adv Sci (Weinh). 2024 Jun 19;11(31):2403093. doi: 10.1002/advs.202403093 (PMC11336976; doi:10.1002/advs.202403093)
Supplement: Supplementary file 1 — Supporting Information [file ADVS-11-2403093-s001.docx]

**Title:** CKB Promotes Mitochondrial ATP Production by Suppressing Permeability Transition Pore

**Authors and Corresponding Author***

Le He, Jianghua Lin, Shaojuan Lu, Hao Li, Jie Chen, Xinyi Wu, Qixin Yan, Hailiang Liu*, Hui Li*, Yufeng Shi*

**Affiliations**

Le He, Jianghua Lin, Shaojuan Lu, Hao Li, Jie Chen, Xinyi Wu, Qixin Yan, Hui Li, Hailiang Liu, Yufeng Shi

Tongji University Cancer Center, Shanghai Tenth People’s Hospital of Tongji University, School of Medicine, Tongji University, Shanghai 200092, China

E-mail: yshi@tongji.edu.cn; hli009@tongji.edu.cn; hailiang_1111@tongji.edu.cn

Yufeng Shi

Key Laboratory of Spine and Spinal Cord Injury Repair and Regeneration of Ministry of Education, Tongji University Cancer Center, Shanghai Tenth People’s Hospital of Tongji University, School of Medicine, Tongji University, Shanghai 200092, China

Hailiang Liu

State Key Laboratory of Cardiology and Medical Innovation Center, Shanghai East Hospital, School of Medicine, Tongji University, Shanghai, 200123, China


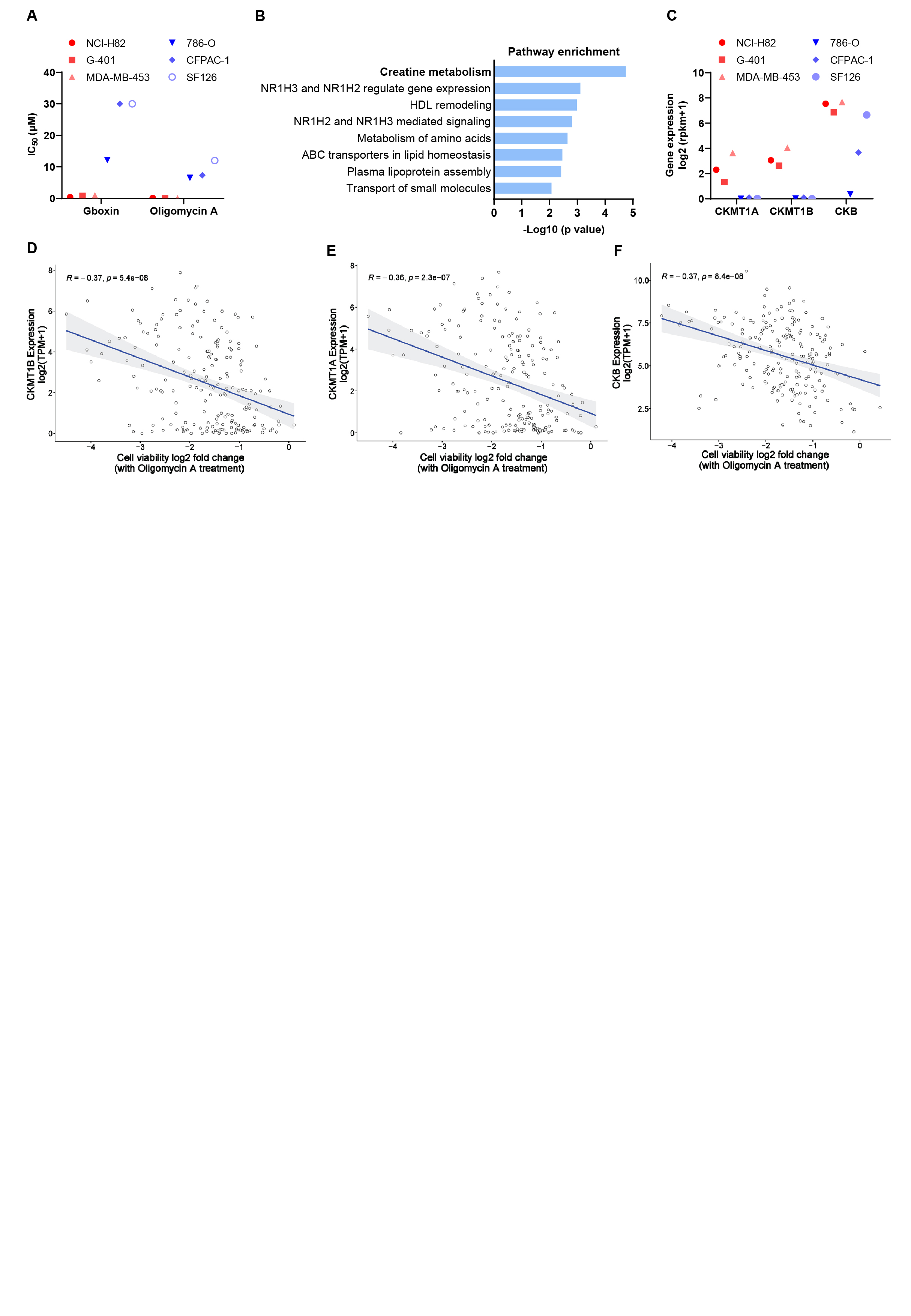


**Figure S1.** Upregulation of creatine kinases in cells sensitive to F1F0 ATP synthase inhibition. A) Determination of half inhibitory concentration (IC_50_) for cancer cell lines sensitive (NCI-H82, G-401 and MDA-MB-453) or resistant (786-O, CFPAC-1, and SF126) to F1F0 ATP synthase inhibitors, Gboxin and Oligomycin A. Cell viability analysis was conducted to establish the IC_50_ of cancer cell lines to Gboxin or Oligomycin A. Cells were treated with compounds for 3 days. B) KEGG enrichment analysis of upregulated genes in Gboxin-sensitive (NCI-H82, G-401, and MDA-MB-453) cell lines compared to resistant (786-O, CFPAC-1, and SF126) cell lines reveals that the most upregulated genes in Gboxin-sensitive cancer cells are involved in creatine metabolism. C) Relative expression of genes, CKMT1B, CKMT1A, and CKB in Gboxin-sensitive (NCI-H82, G-401, and MDA-MB-453) and resistant (786-O, CFPAC-1, and SF126) cell lines. Gene expression data is extracted from the Cancer Dependency Map Project (Depmap) database. D to F) Positive correlation of CKMT1B (D), CKMT1A (E), and CKB (F) expression with cell sensitivity to Oligomycin A treatment at 2.4 nM. Data on gene expression and sensitivity to Oligomycin A treatment for 200 cancer cell lines were extracted from the Depmap.


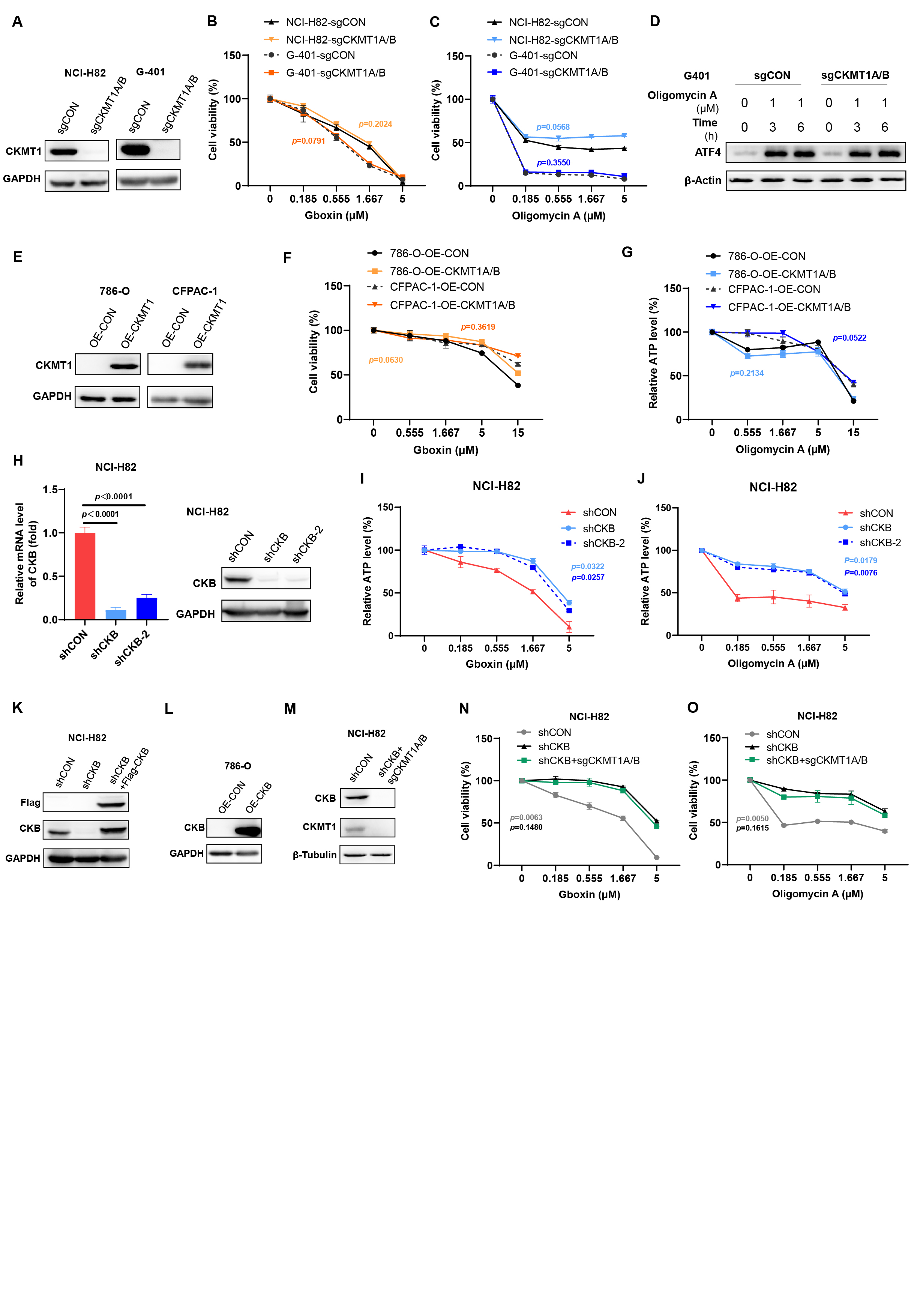


**Figure S2.** Impact of CKMT1 knockout on cell sensitivity to F1F0 ATP synthase inhibition. A) Immunoblot analysis shows knockout of CKMT1 in NCI-H82 and G-401 cells transfected with CKMT1A/B-target sgRNA (sgCKMT1A/B) compared to those transfected with vector (sgCON). n = 3. B and C) Cell viability analysis reveals no sensitivity difference to treatment with Gboxin or Oligomycin A between NCI-H82-sgCON and NCI-H82-sgCKMT1A/B cells or G-401-sgCON and G-401-sgCKMT1A/B cells. Cells were incubated with Gboxin (B) and Oligomycin A (C) at various concentrations for 3 days. Mean ± SD. n = 3. The *p* value was calculated by paired *t*-test. D) Western blot analysis demonstrates knockout CKMT1A/B in G-401 cells did not affect ATF4 expression. Cells were treated with DMSO or Oligomycin A (1 μM) for 3 or 6 hours. With β-Actin serving as a loading control, n = 3. E) Immunoblot analysis of CKMT1 expression in 786-O and CFPAC-1 cells transfected with vector (OE-CON) or CKMT1A/B (OE-CKMT1). n = 3. F and G) Cell viability analysis shows no sensitivity difference to the treatment of Gboxin or Oligomycin A between 786-O-OE-CON and 786-O-OE-CKMT1A/B cells or CFPAC-1-OE-CON and CFPAC-1-OE-CKMT1A/B cells. Cells were incubated with Gboxin (F) and Oligomycin A (G) at various concentrations for 3 days. Mean ± SD. n = 3. The *p* value was calculated by paired *t*-test. H) RT-qPCR (left) and immunoblot (right) analysis for CKB expression in NCI-H82 cells transfected with vector (shCON) or CKB-targeting shRNA (shCKB and shCKB-2). Mean ± SD. n = 3. The *p* value was calculated by paired *t*-test. I and J) Cell viability analyses demonstrate the critical role of CKB in maintaining cell sensitivity to the inhibition of F1F0 ATP synthase. The Cell Titer-Glo reagent was used to detect the cell viability of NCI-H82-shCON, NCI-H82-shCKB and NCI-H82-shCKB-2 incubated with Gboxin (I) or Oligomycin A (J) at various concentrations for 3 days. Mean ± SD. n = 3. Paired *t*-test reveals the lowest *p* value compared to the vector. K) Western blot analyses confirm the re-expression of CKB in NCI-H82-shCKB cells. NCI-H82-shCKB cells were infected with a virus expressing an empty vector (shCKB) or Flag-CKB (shCKB+Flag-CKB) for 3 days before analysis. n = 3. L) Immunoblot analysis of CKB expression in 786-O cells transfected with vector (OE-CON) or CKB cDNA (OE-CKB). n = 3. M) Immunoblot analysis confirms the knockout of CKMT1 and CKB in CKB/CKMT1 double-null NCI-H82 cells (shCKB+sgCKMT1A/B). n = 3. N and O) Cell viability analyses show no sensitivity difference to treatment with Gboxin or Oligomycin A between NCI-H82-shCKB+sgCKMT1A/B and NCI-H82-shCKB cells. Cells were incubated with Gboxin (N) and Oligomycin A (O) at various concentrations for 3 days. Mean ± SD. n = 3. The *p* value was calculated by paired *t*-test.


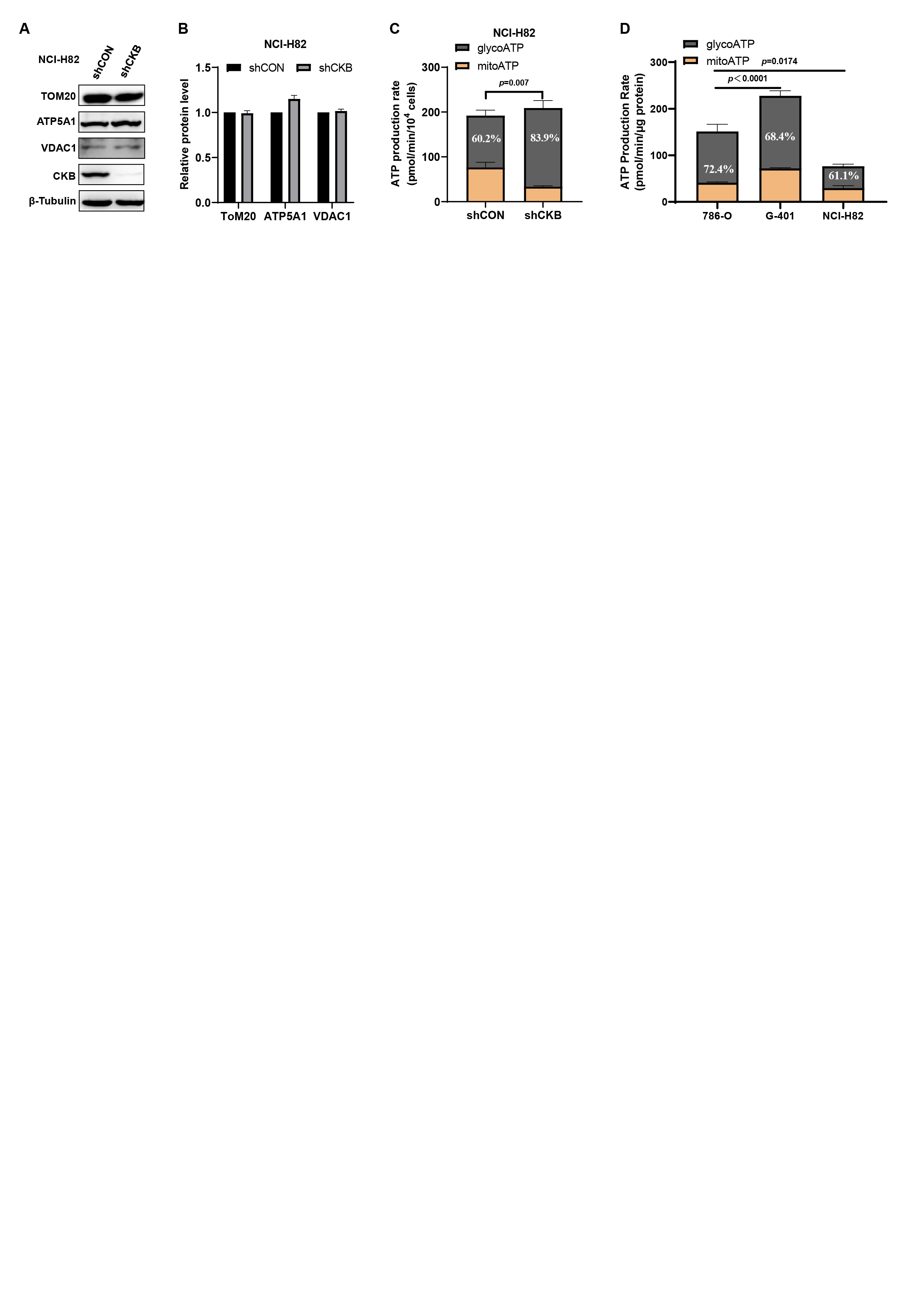


**Figure S3.** CKB depletion does not affect mitochondrial housekeep proteins expression. A and B) Western blot analysis reveals no significant difference in the expression of mitochondrial housekeeping proteins (ATP5A1, VDAC1, and TOM20) in cells deficient in CKB. NCI-H82 cells were infected with a virus expressing control shRNA (shCON) or CKB shRNA (shCKB) as indicated. n = 3. (B) Quantification of (A). Mean ± SD; n = 3. C) XF real-time ATP rate assay shows reduced mitochondrial ATP production but an increased glycolytic ATP production rate in NCI-H82-shCKB cells compared to those in NCI-H82-shCON cells, with total ATP production remaining unchanged. Mean ± SD; n = 3. The *p* value was calculated by paired *t*-test. D) XF real-time ATP rate assay shows higher mitochondrial ATP production rate in Gboxin-sensitive cells (NCI-H82 and G-401) compared to that in Gboxin-resistant 786-O cells. Mean ± SD; n = 3. The *p* value was calculated by paired *t*-test.


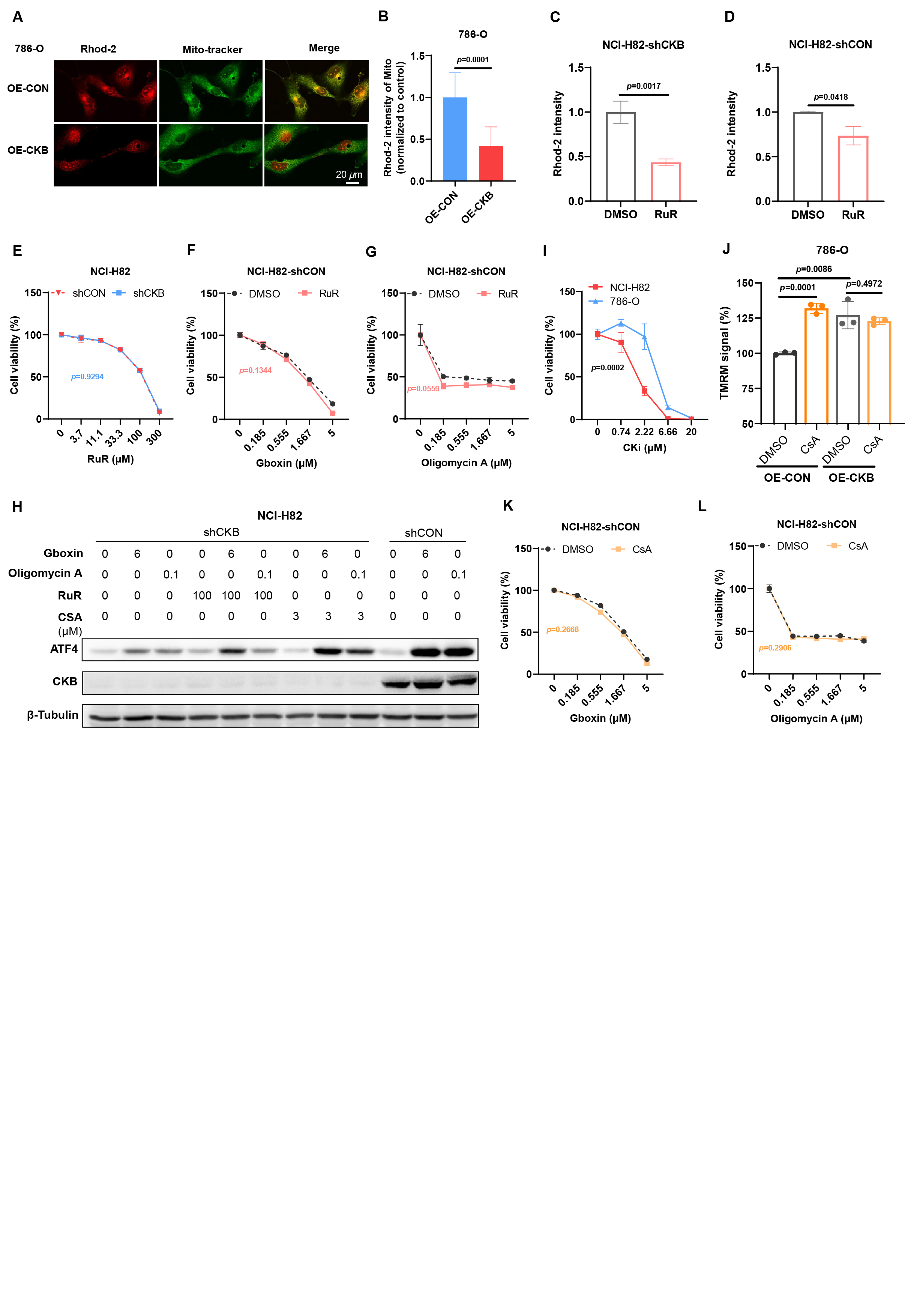


**Figure S4.** Impact of CKB depletion on mCa^2+^ levels, and cell sensitivity to MCU, mPTP, and F1F0 ATP synthase inhibitors alone and in combination. A and B) Confocal microscope images demonstrate decreased mCa^2+^ in 786-O-OE-CKB cells compared to that in 786-O-OE-CON cells. Rhod-2 probe (5 μM) was used to detect mCa^2+^, followed by incubation with 200 nM Mito-tracker Green. (B) quantification of (A). Mean ± SD; n = 3. The *p* value was calculated using a paired *t*-test. C and D) Rhod-2 staining shows that Ruthenium red (RuR) treatment decreases mCa^2+^ levels in both NCI-H82-shCKB (shCKB) cells (C) and NCI-H82-shCON (shCON) cells (D). Cells were pretreated with the mCa^2+^ uniporter (MCU) inhibitor RuR (100 μM, 30 mins) and then incubated with 5 μM Rhod-2 for another 30 mins before analysis. Mean ± SD; n = 3. The *p* value was calculated by paired *t*-test. E) Cell viability analysis shows no significant difference between NCI-H82-shCON and NCI-H82-shCKB cells in response to RuR treatment. Cells were treated with various concentrations of RuR for 3 days before analysis. Mean ± SD; n = 3. The *p* value was calculated by paired *t*-test. F and G) Cell viability analysis demonstrates no sensitivity alteration to the treatment of Gboxin (F) or Oligomycin (G) with NCI-H82-shCON (shCON) cells in the presence or absence of RuR. Cells were pretreated with 100 μM RuR for 30 mins, and then incubated with Gboxin (F) and Oligomycin (G) at various concentrations for another 3 days before analysis. Mean ± SD; n = 3. The *p* value was calculated by paired *t*-test. H) Western blot analysis of ATF4 demonstrates pre-incubation of RuR or Cyclosporin A (CsA) sensitized NCI-H82-shCKB cells to the treatment of Gboxin or Oligomycin A. Cells were pretreated with or without 100 μM RuR or 3 μM CsA for 30 minutes, followed by incubation of Gboxin and Oligomycin A for 3 h. n = 3. I) The effect of CKB inhibitor (CKi) on the viability of NCI-H82 and 786-O cells. cells treated with a series concentration of CKi for 3 days before analysis. J) Treatment of Cyclosporin A (CsA, 3 μM, 30 min.) increases MMP in 786-O-OE-CON cells but not that in 786-O-OE-CKB cells. MMP was detected by TMRM staining. Mean ± SD; n = 3. The *p* value was calculated using a paired *t*-test. K and L) Cell viability analysis demonstrates no sensitivity alteration to the treatment of Gboxin (K) or Oligomycin (L) with NCI-H82-shCON (shCON) cells in the presence or absence of mPTP inhibitor Cyclosporin A (CsA). Cells were pretreated with 3 μM CsA for 30 mins, and then incubated with Gboxin (K) and Oligomycin (L) at various concentrations for another 3 days before analysis. Mean ± SD; n = 3. The *p* value was calculated by paired *t*-test.


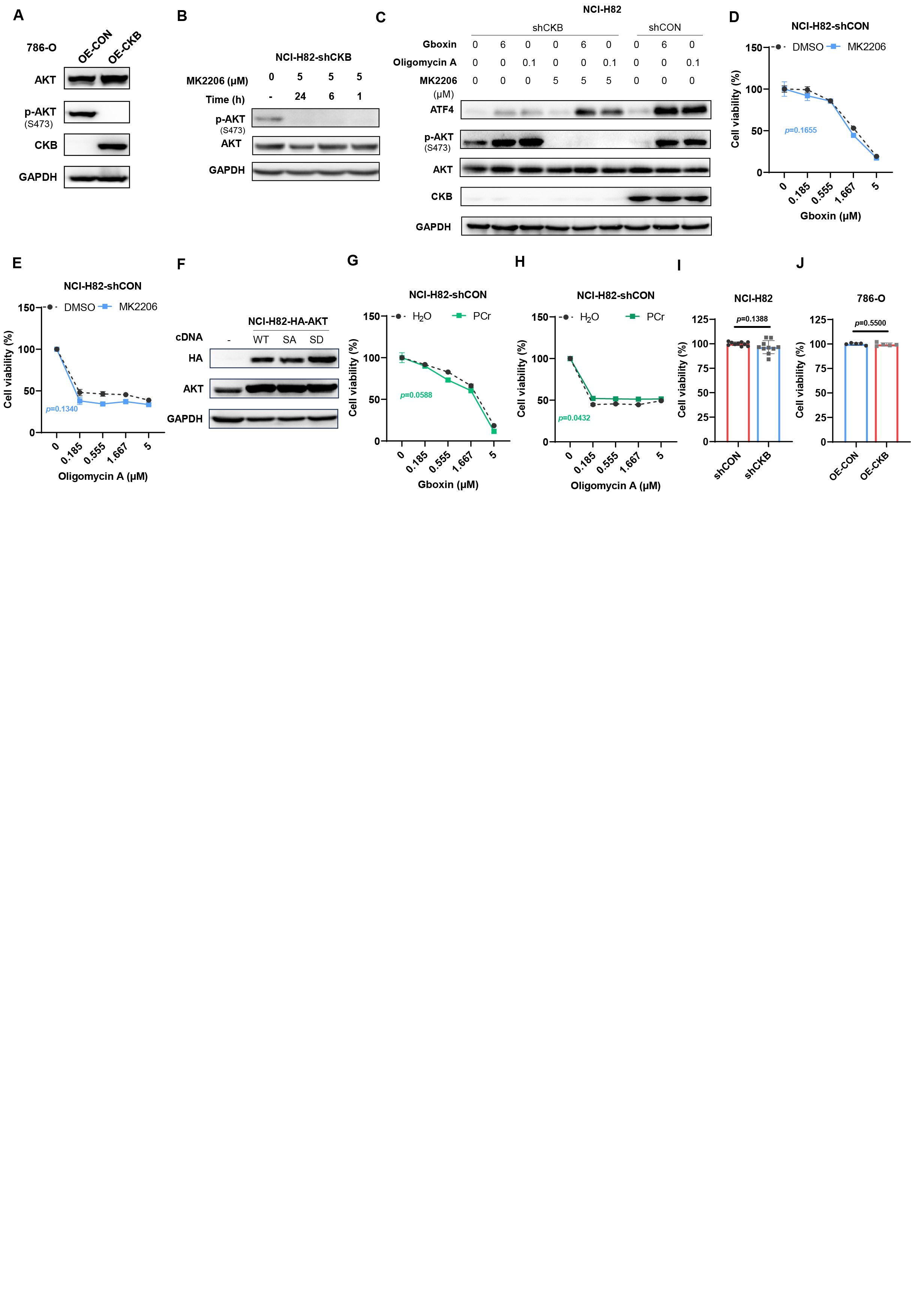


**Figure S5.** CKB and Phosphocreatine Suppress AKT Activation. A) Western blot analysis reveals decreased AKT activation upon CKB overexpression in 786-O cells. n = 3. B) Western blot analysis shows that treatment with MK2206 (5 μM) inhibits AKT activation in NCI-H82-shCKB cells. Cells were treated with or without MK2206 for indicated time before analysis. n = 3. C) Western blot analysis of ATF4 expression demonstrates MK2206 treatment sensitizes NCI-H82-shCKB cells to the inhibition of F1F0 ATP synthase by Gboxin and Oligomycin A. Cells were pretreated with or without 5 μM MK2206 for 1 h, followed by incubation of Gboxin and Oligomycin A for anther 3 h. n = 3. D and E) Cell viability analysis reveals no sensitivity alteration to the treatment of Gboxin or Oligomycin A in NCI-H82-shCON cells in the presence or absence of the AKT inhibitor MK2206. Cells were incubated with 5 μM MK2206 for 30 mins, and then treated with Gboxin (D) or Oligomycin A (E) at various concentrations for another 3 days before analysis. Mean ± SD; n = 3. The *p* value was calculated by paired *t*-test. F) Western blot analyses of AKT and HA-tagged proteins in NCI-H82 cells transfected with a vector expressing wildtype AKT (HA-AKT(WT)), phosphorylation-resistant mutant AKT (HA-AKT(SA)), or phosphorylation-mimicking mutant AKT (HA-AKT(SD)). n = 3. G and H) Cell viability analysis shows no sensitivity alteration to the treatment of Gboxin or Oligomycin A in NCI-H82-shCON cells in the presence or absence of phosphocreatine (PCr). Cells were pretreated with 10 mM PCr for 1 hour, and then incubated with Gboxin (G) and Oligomycin A (H) at various concentrations for another 3 days before analysis. Mean ± SD; n = 3. The *p* value was calculated by paired *t*-test. I and J) Cell viability analysis shows that CKB depletion in NCI-H82 cells (I) or CKB overexpression in 786-O cells (J) does not have significant effects on cell growth rate, n = 10 or 5. The *p* value was calculated by paired *t*-test.


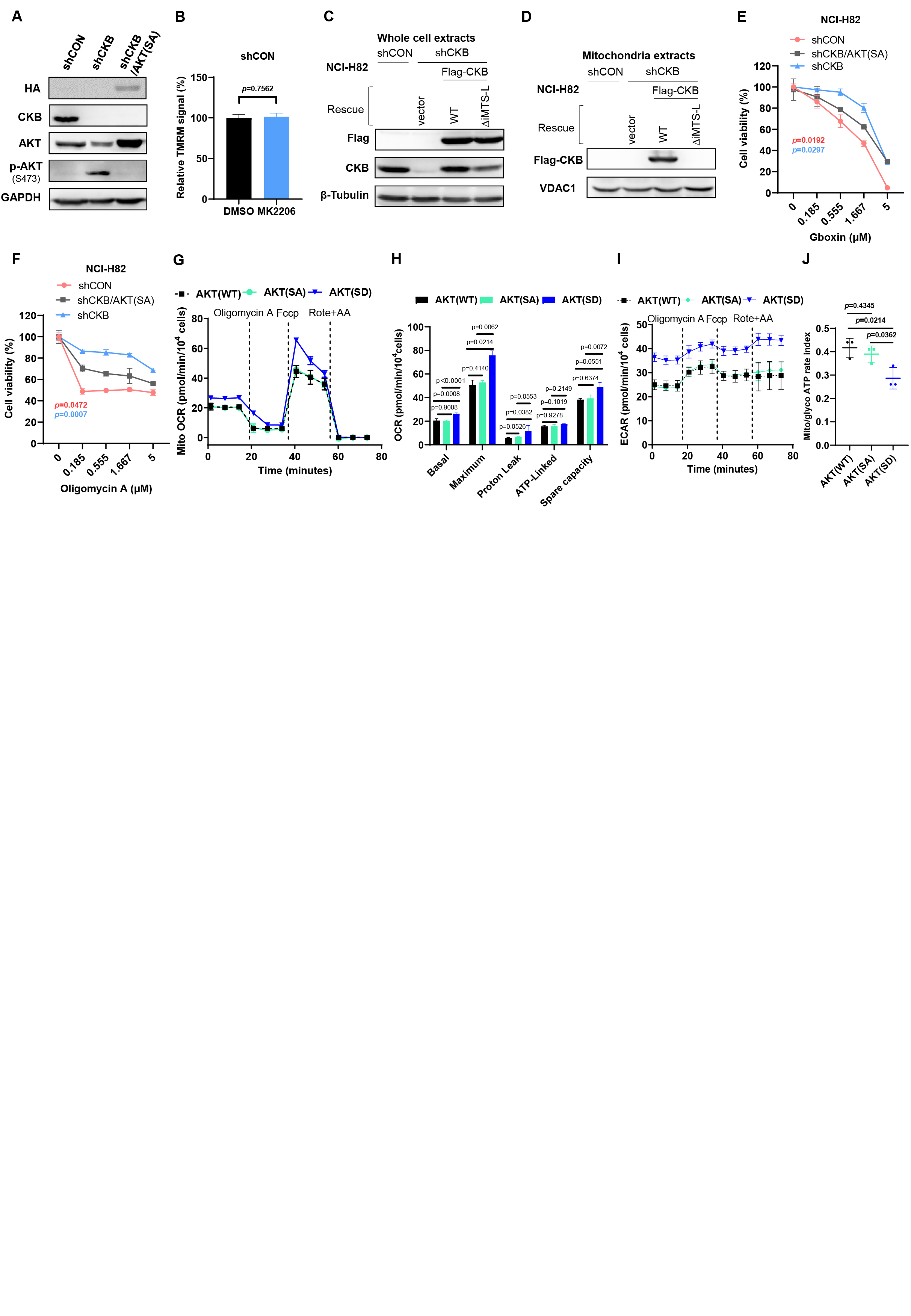


**Figure S6.** CKB suppresses AKT activation, ensuring efficient mitochondrial ATP production. A) Western blot analyses show that expression of a phosphorylation-resistant mutant (AKT(SA)) inactivates endogenous AKT in NCI-H82-shCKB cells. n = 3. B) Treatment with the AKT inhibitor MK2206 (5 μM, 30 mins) shows no detectable alteration in mitochondrial membrane potential (MMP) in NCI-H82-shCON cells. Mean ± SD; n = 3. The *p* value was calculated by paired *t*-test. C) Western blot analysis of CKB exprssion with whole cell extracts (C) or mitochondria extracts (D) of NCI-H82-shCON cells or NCI-H82-shCKB cells expressing Flag-CKB(WT) or Flag-CKB(ΔiMTS-L). n = 3. E and F) Cell viability analysis shows that expression of AKT(SA) sensitizes NCI-H82-shCKB cells to the inhibition of F1F0 ATP synthase by Gboxin (E) or Oligomycin A (F). Cells were treated with various concentrations of Gboxin or Oligomycin A for 3 days before cell viability analysis was performed. Mean ± SD; n = 3. The *p* value was calculated by paired *t*-test. G and H) Seahorse analyzer assays show that expression of AKT(SD), but not AKT(SA) or AKT(WT), increased basal, maximum, and proton leakage induced oxygen consumption rate (OCR) in NCI-H82 cells. Cells were treated with Oligomycin A (18 min.), FCCP (36 min.), and a mixture of Rotenone and Antimycin A (Rote/AA, 54 min.). Mean ± SD; n = 3. (H) Quantification of (G). Mean ± SD; n = 3. The *p* value was calculated by paired *t*-test. I) Seahorse analyzer assays show that expression of AKT(SD) increased glycolytic activity compared to that in NCI-H82 cells expressing AKT(SA) or AKT(WT). Cells were treated with Oligomycin A (18 min.), FCCP (36 min.), and a mixture of Rotenone and Antimycin A (Rote/AA, 54 min.). Mean ± SD; n = 3. The *p* value was calculated by paired *t*-test. J) XF real-time ATP rate assay shows expression of AKT(SD) decreases mitochondrial (mito)/glycolytic (glyco) ATP production rate compared with expression of AKT(SA) or AKT(WT) in NCI-H82 cells. Mean ± SD; n = 3. The *p* value was calculated by paired *t*-test.

**
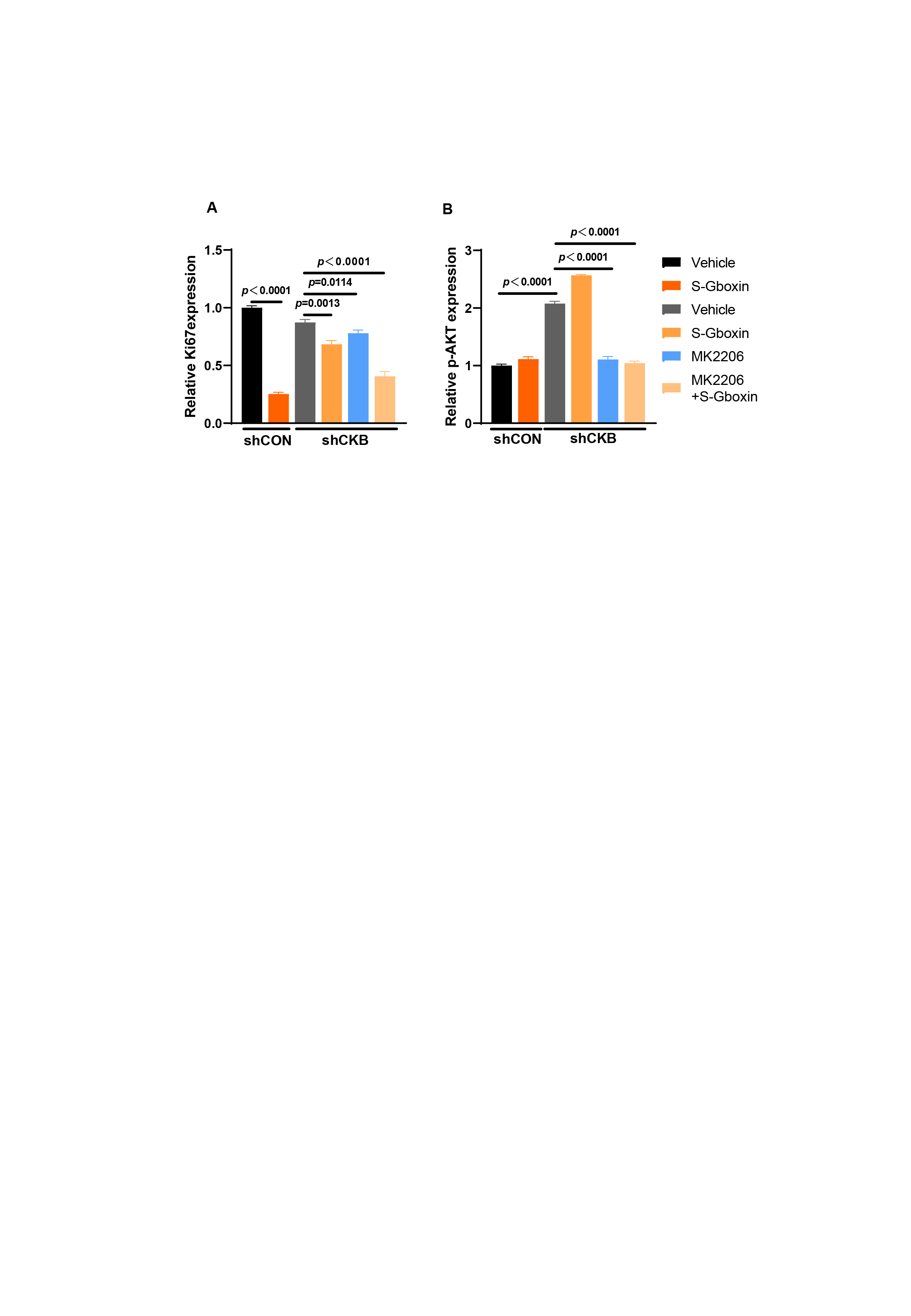
**

**Figure S7.** Quantification of Ki67 (A) and p-AKT expression (B) of Figure 6D. n = 3. The *p* value was calculated by paired *t*-test.
